# Neural field models with transmission delays and diffusion

**DOI:** 10.1186/s13408-020-00098-5

**Published:** 2020-12-09

**Authors:** Len Spek, Yuri A. Kuznetsov, Stephan A. van Gils

**Affiliations:** 1 Department of Applied Mathematics, University of Twente, Enschede, The Netherlands; 2 Department of Mathematics, Utrecht University, Utrecht, The Netherlands

**Keywords:** Neural field, Delay equation, Sun-star calculus, Hopf bifurcation, Normal form, Numerical bifurcation analysis

## Abstract

A neural field models the large scale behaviour of large groups of neurons. We extend previous results for these models by including a diffusion term into the neural field, which models direct, electrical connections. We extend known and prove new sun-star calculus results for delay equations to be able to include diffusion and explicitly characterise the essential spectrum. For a certain class of connectivity functions in the neural field model, we are able to compute its spectral properties and the first Lyapunov coefficient of a Hopf bifurcation. By examining a numerical example, we find that the addition of diffusion suppresses non-synchronised steady-states while favouring synchronised oscillatory modes.

## Introduction

In the study of neurological disease, non-invasive imaging techniques are often used to get an understanding of the structure and functioning of the brain on intermediate scales. As they give a course-grained view of the neuronal activity, mean-field models are a natural fit to describe the observed dynamics [1, 2]. In this paper we use a neural field model with gap-junctions, electrical connections between neurons, which are thought to be related to observed synchronisation of neural tissue in Parkinson’s disease [3, 4]. We study the effect of gap junctions on the dynamics of the model. We mainly focus on the stability of steady-states, periodic oscillations and the bifurcations which lead to a qualitative change in behaviour.

To properly address the difference in time-scales between gap-junctions and synaptic connections, we use a neural field with transmission delays for the synaptic connections. This leads to a complicated model which is infinite-dimensional and has spatially-distributed delays. The dynamical theory for such models is not readily available. In this paper, we address the analytic problems which arise from these abstract delay differential equations.

We use the sun-star calculus as the basic functional analytic tool to cast the equation in the variation-of-constants form. We exploit the results by Janssens [5, 6] that allow the linear part of the equation, without the delays, to be unbounded, as is the case for the diffusion operator.

### Background

Neural field models try to bridge the gap between single neurons models [7] and whole brain models [8] by modelling the qualitative behaviour of large groups of neurons. In the seminal work of Wilson and Cowan [9, 10], they modelled two populations of excitatory and inhibitory neurons and analysed the dynamical properties of the resulting model. A neural field uses spatial and temporal averaging of the membrane voltage of a population of neurons. The synaptic connections are modelled by a convolution of a connectivity kernel and a nonlinear activation function. This leads to a set of two integro-differential equations with delays.

These models have been simplified by Amari [11] by combining the excitatory and inhibitory populations into a single population and made more realistic by Nunez [12] by including transmission delays. These delays arise from the finite propagation speed of action potentials across an axon and the delay due to dendritic integration. There has been considerable interest in the role of these delays in the spatiotemporal dynamics [13–21]. Further modelling work by Coombes, Venkov and collaborators show the usefulness of these neural fields for understanding neural activity [22–25].

Roxin and collaborators first did a bifurcation analysis for neural fields with a single fixed delay [26–28]. Faugeras and collaborators investigated the stability properties of stationary solutions of these neural fields with distance dependent delays [29–32] using a functional analytic approach based on formal projectors. In [33] it was shown that the neural fields can be studied as abstract delay differential equations to which the sun-star framework can be applied. They used this to compute normal form coefficients for bifurcations of equilibria. Dijkstra et al. [34] expanded their analysis to Pitchfork–Hopf bifurcations, and Visser et al. [35] analysed a neural field with delays on a spherical domain. We build on [33, 34] by introducing gap-junctions into the neural field model and studying the resulting bifurcations and dynamics.

Gap-junctions are electrical connections between neurons, which directly exchange ions through a connexin-protein. This is in contrast to synaptic connections, where a potential is induced across the synapse by neurotransmitters. These gap-junctions are thought to be related to Parkinson’s disease by synchronising neurons in the *globus pallidus* [3, 4]. Gap-junctions can be modelled as a simple diffusion process [24]. There have been some attempts to incorporate gap-junctions into networks of coupled neurons [36–38], but to our knowledge not yet within a proper neural field model.

### Theoretical framework

As mentioned before, we use the sun-star calculus for delay differential equations to formally analyse these neural field models with transmission delays. This mathematical theory for delay differential equations was constructed by Diekmann et al., see [39] and the references therein. This theory uses the space $X^{\odot }$, pronounced *X*-sun, which is the largest subspace of strong continuity of the adjoint semigroup. It allows us to employ the classical Fredholm alternative, which plays a key role in the computation of the normal form coefficients. As a result, many of the mathematical techniques developed for the analysis of ODEs, such as the centre manifold reduction and the Hopf bifurcation theorem, can be generalised for these abstract delay differential equations.

Recently, Janssens [5, 6] has begun expanding the sun-star calculus to the case where the linear part, which contains no delays, is an unbounded operator. This allows us to study both the neural field with and without diffusion in the same framework. This unifying theory then allows us to fill in the gap in the proofs of [33], while obtaining the same results for a neural field with diffusion.

There are also other theoretical frameworks possible. The first approach to develop a geometric theory for delay equations along the lines of ODEs was proposed by Hale [40] who used formal adjoint operators. Formal adjoint operators were also used by Faria and Magalhaes [41–43] to study Hopf and Bogdanov–Takens bifurcations. Wu [44] used the formal adjoint method to study reaction-diffusion systems with delays and prove the necessary theorems for bifurcation analysis.

There is a difference whether to take as a starting point an abstract integral equation, like we do, or an abstract ODE like in the integrated semigroup approach [45–47]. Integrated semigroups have been used to deal with classical delay differential equations as abstract ODEs with non-dense domains. By classical we here mean that the state space is $\mathbb {R}^{n} $. In the case of the neural field equations we consider, the state space is an abstract Banach space. It might very well be possible that the formalism of integrated semigroups is general enough to cover this as well, but as far as we know, this has not been done as yet. We prefer the sun-star formalism as it allows us to work with the variation-of-constants formula in the state space *X*, albeit after an excursion in the bigger space $X^{\odot \ast }$. In addition, the projectors are based on duality pairing and the classical Fredholm alternative, while in the integrated semigroup formalism the projectors are based on a formal inner product [48].

There are also two approaches to compute normal form coefficients. In the first approach, the abstract ODE is split into a finite dimensional and an infinite dimensional one. By decoupling these step by step, the centre manifold is rectified and the equation on it is normalised [45–47]. In the second approach, which we follow, we parametrise the centre manifold and assume that the finite dimensional ODE on it is in normal form. As the delay differential equation has an abstract state space, this ODE is also an abstract ODE. The Taylor coefficients of the centre manifold are obtained in a step-by-step procedure that simultaneously gives us the coefficients of the normal form [49, 50]. In this way, the sun-star calculus approach leads to explicit, compact and easy to evaluate expression for the normal form coefficients [51]. These coefficients are obtained using the true duality pairing, for which the classical Fredholm alternative holds. Of course, the resulting formulas are equivalent, but the approach we adopted is more straightforward.

In the sun-star calculus we choose to model the neural field as a continuous function in space. In [29] and [31] the authors instead choose to use the $L^{2}$-functions, based on the work in [52–54]. This leads to some mathematical complications dealing with the smoothness of the nonlinearity, as laid out previously in Sect. 2.4 of [55]. This was later rectified in [56]. Moreover, from a physiological point of view, it is not clear why the potential of the neural field should be merely square integrable, instead of continuous.

Finally, we want to briefly comment on the need of a theoretical framework to study these neural fields. Software packages, such as DDE-BIFTOOL [57], can perform numerical bifurcation analysis of delay equations. However, they cannot directly be applied to these delayed integro-differential equations. While a discretised model can be studied with these software packages, there is no guarantee that the dynamical properties converge to those of the full neural field. In this work, the formulas of the normal form coefficients are exact and can be evaluated to arbitrary precision.

In this paper we build on the work of Janssens [5, 6] and prove the necessary theorems to use the sun-star calculus to study our neural field model with diffusion and without diffusion. We then derive the spectrum and resolvent of a neural field with delays, diffusion and a connectivity kernel of a sum of exponentials. Finally, we compute the first Lyapunov coefficient of a Hopf bifurcation and verify our results by simulating the full neural field numerically.

### Modelling

In this section we derive the neural field model with transmission delays and gap junctions. This is largely based on a derivation by Ermentrout and Cowan [20].

We start with a collection of neurons $i=1,2,3,\ldots $ and denote the (somatic) potential of neuron *i* at time *t* by $u_{i}(t)$ and its firing rate by $f_{i}(t)$. We assume that there is a nonlinear dependence of $f_{i}$ on $u_{i}$ given by

$$ f_{i}(t) = S_{i}\bigl(u_{i}(t)\bigr). $$

We define $\Phi _{i,j}(t)$ to be the postsynaptic potential appearing on postsynaptic cell *i* due to a single spike from presynaptic cell *j*. We assume a linear summation of the postsynaptic potentials, so the total potential received at the soma due to the synaptic connection between cell *i* and *j* can be modelled as

$$ G_{i,j}(t)= \int _{-\infty }^{t} \Phi _{i,j}(t-s)f_{j}(s- \tau _{i,j})\,ds, $$

where $\tau _{i,j}$ is the delay due to the finite propagation speed of action potentials along an axon and other factors such as dendritic integration. We define $\Psi _{i}(t)$ to be the potential appearing in neuron *i* due to a gap-junction current $I_{i,\mathrm{gap}}(t)$. The resulting model for $u_{i}$ becomes

1 $$ u_{i}(t) = \Psi _{i}(t) + \sum _{j} \int _{-\infty }^{t} \Phi _{i,j}(t-s)S_{j} \bigl(u_{j}(s- \tau _{i,j})\bigr)\,ds. $$

We can reduce this integral equation if we have a model for Φ and Ψ. For cell *i*, let us consider a passive membrane with a time constant $1/\alpha _{i}$, a resistance $R_{i}$ and an injected postsynaptic current $I_{i,j,\mathrm{syn}}(t)$

$$ \frac{1}{\alpha _{i}} \frac{d \Phi _{i,j}}{dt} + \Phi _{i,j} = R_{i} I_{i,j,\mathrm{syn}}(t) $$

and similarly when a gap-junction current is injected

$$ \frac{1}{\alpha _{i}} \frac{d \Psi _{i}}{dt} + \Psi _{i} = R_{i} I_{i,\mathrm{gap}}(t). $$

If we now apply the Laplace transform $\mathcal{L}$ to equation (1), we get

$$ \biggl(\frac{s}{\alpha _{i}}+1 \biggr)\mathcal{L}(u_{i}) (s) = R_{i} \mathcal{L}(I_{i,\mathrm{gap}}) (s) + R_{i} \sum_{j} \mathcal{L}(I_{i,j,\mathrm{syn}}) (s) \mathcal{L}\bigl(S_{j}\bigl(u_{j}(\cdot -\tau _{i,j})\bigr)\bigr) (s). $$

We assume that the synaptic dynamics are dominated by the time-scale of the membrane. This means we can reduce $I_{i,j,\mathrm{syn}}(t)$ to $w_{i,j} \delta (t)$, where *δ* is the Dirac-delta distribution and $w_{i,j}$ represents the strength of the synaptic connection, where a negative value corresponds to inhibition. Taking the inverse Laplace transform results in a system of differential equations

2 $$ \biggl(\frac{1}{\alpha _{i}} \frac{d}{dt}+1 \biggr) u_{i}(t) = R_{i} I_{i,\mathrm{gap}}(t) + R_{i} \sum_{j} w_{i,j} S_{j}\bigl(u_{j}(t-\tau _{i,j})\bigr). $$

We want to model this network of cells by a neural field. Suppose we have a sequence of similar neurons $i=1,2,\ldots , M$ on the interval $\Omega =[-1,1]$ and we model the gap-junctions as a simple resistor between adjacent neurons, we arrive at the formula

3 $$ \biggl(\frac{1}{\alpha } \frac{d}{dt}+1 \biggr) u_{i}(t) = R g \bigl(u_{i-1}(t) - 2 u_{i}(t) + u_{i+1}(t)\bigr) + R \sum _{j} w_{i,j} S\bigl(u_{j}(t-\tau _{i,j})\bigr). $$

We will now take the limit as $M \rightarrow \infty $, while scaling *g* by $M^{2}$ and $w_{i,j}$ by $1/M$, to find our neural field model

4 $$ \frac{\partial u}{\partial t}(t,x)= d \frac{\partial ^{2} u}{\partial x^{2}}(t,x) - \alpha u(t,x)+ \alpha \int _{\Omega }J\bigl(x,x'\bigr)S\bigl(u\bigl(t- \tau \bigl(x,x'\bigr),x'\bigr)\bigr)\,dx'. $$

We have not specified yet what happens with the gap-junctions at the boundary of our domain. It is natural to assume that no current leaks away at the boundaries, which corresponds to Neumann boundary conditions in the neural field

$$ \frac{\partial u}{\partial x}(t,\pm 1) =0. $$

### Overview

This paper is divided into three parts, each of which can mostly be read independently.

In Sect. 2, we construct the sun-star calculus for abstract delay differential equations and derive the variation-of-constants formula. In particular we prove a novel characterisation for sun-reflexivity. Furthermore we consider linearisation, the corresponding spectrum and a normal form derivation for Hopf bifurcation of the nonlinear equations. In appendix A we elaborate on the case when the unbounded linear operator is the diffusion operator. We expect the reader to be familiar with the basics of the sun-star framework in the book by Diekmann et al. [39].

In Sect. 3 we derive formulas for the eigenvalues and eigenvectors for a neural field with a connectivity defined by a sum of exponentials. We also explicitly construct the solution to the resolvent problem for this class of neural field models.

In Sect. 4 we do a numerical study for a neural field model with specific parameter values. We compute the first Lyapunov coefficient for the Hopf bifurcation and investigate how it is influenced by the diffusion term. We also investigate the emergence of periodic behaviour using numerical simulations of the neural field.

## Abstract delay differential equations in the sun-star framework

In this section we first develop the sun-star calculus for a large class of abstract delay differential equations (ADDE). This leads to a variation-of-constants formulation of (ADDE). Next we study the linearisation and obtain results on the spectrum. Finally, we construct a method for computing the first Lyapunov coefficient for a Hopf bifurcation of nonlinear equations. We build on the theory developed by Janssens [5], who considers a class of abstract delay differential equations with a possibly unbounded linear part.

Consider two Banach spaces *Y* and $X = C([-h,0];Y)$ over $\mathbb{R}$ or $\mathbb{C}$. Let *S* be a strongly continuous semigroup on *Y* with its generator *B*, and let $G: X\rightarrow Y$ be a (nonlinear) globally Lipschitz-continuous operator. Note that the assumption that the semigroup *S* is compact is not necessary, in contrast to what is assumed by Wu [44].

We introduce now our main object of study:

ADDE $$ \textstyle\begin{cases} \dot{u}(t)=B u(t)+G(u_{t}), \\ u_{0}=\varphi \in X. \end{cases} $$

Here $u_{t} \in X$, where $u_{t}(\theta )= u(t+\theta )$ for $t\geq 0$ and $\theta \in [-h,0]$.

In the remaining sections we are mainly interested in the case where *B* is a diffusion operator acting in the space of continuous functions $Y=C([-a,a];\mathbb{R})$. We have summarised the relevant properties of the diffusion operator in Appendix A. However, the theorems which are proven in this section hold for any operator *B* that generates a strongly continuous semigroup *S* on *Y*. This fills in some technical details missing in [33], where $B=-\alpha I$, which does not generate a compact semigroup.

On *X* we consider the strongly continuous semigroup $T_{0}$ defined by

5 $$ \bigl(T_{0}(t)\varphi \bigr) (\theta ):= \textstyle\begin{cases} \varphi (t+\theta ) &t+\theta \in [-h,0], \\ S(t+\theta )\varphi (0) &t+\theta >0. \end{cases} $$

Here $\varphi \in X$, $t\geq 0$ and $\theta \in [-h,0]$. This semigroup is related to the problem for $G\equiv 0$, i.e.

6 $$\begin{aligned} \textstyle\begin{cases} \dot{v}(t)=B v(t) &\text{for } t>0, \\ v_{0}=\varphi &\text{for } t \in [-h,0]. \end{cases}\displaystyle \end{aligned}$$

The solution of problem (6) is then given by $v_{t}:=T_{0}(t)\varphi $.

### Lemma 1

([58, Theorem VI.6.1])

*The generator* $A_{0}$ *of the semigroup* ${T_{0}}$ *is given by*

7 $$ A_{0} \varphi = \dot{\varphi },\qquad D(A_{0})=\bigl\{ \varphi \in C^{1}\bigl([-h,0];Y\bigr)| \varphi (0)\in D(B) \textit{ and } \dot{\varphi }(0)=B\varphi (0)\bigr\} . $$

We will interpret (ADDE) as problem (6) with some nonlinear perturbation $G:X\rightarrow Y$ and use a variation-of-constants formula in *X* to obtain results about the perturbed problem, such as normal form coefficients for local bifurcations. As *G* maps *X* into *Y*, we would like to embed *Y* in a natural way into *X*. A naive approach would be to use a delta-function as an embedding. However, this embedding is not bounded, so the domain of $A_{0}$ would not be preserved under perturbation. This is indeed the case, as the rule for extending a function beyond its original domain, i.e. $\dot{\varphi }(0)=B\varphi (0)$, is incorporated in $D(A_{0})$. Hence adding a perturbation to the rule for extension changes the domain of the generator. A way out is to embed this problem into a larger space. A natural choice would be $Y \times X$, where we have a continuous embedding $\ell :Y \rightarrow Y\times \{0\}$, and we can separate the extension and translation part of $A_{0}$ into $Y \times \{0\}$ and $\{0\} \times X$ respectively.

More formally we use the sun-star calculus as developed in the book by Diekmann et al. [39] to construct the space $X^{\odot *}$, which contains the space $Y\times X$. We will first restrict the dual space $X^{*}$ to the sun space $X^{\odot }$, on which $T_{0}^{*}$ is strongly continuous. Then taking the dual we obtain the dual space $X^{\odot *}$. It is convenient to present the relationship of the various spaces schematically in the following ‘duality’ diagram, see Fig. 1.

**Figure 1:**
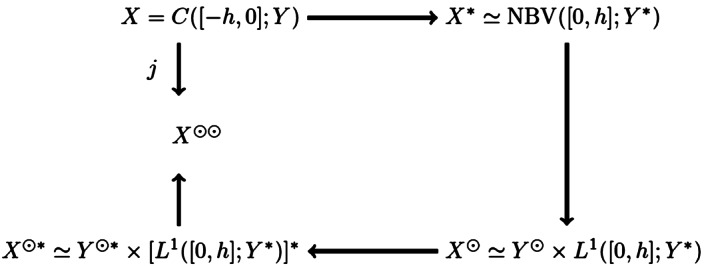
A schematic representation of the various Banach spaces in sun-star calculus [5]

### Characterisation of the sun-dual

Using a generalisation of the Riesz representation theorem, we can find a representation of $X^{*}$, the dual space of *X* [59]. It can be represented as $\operatorname{NBV}([0,h];Y^{*})$, the space of functions $f:[0,h]\rightarrow Y^{*}$ of bounded variation on $[0,h]$, normalised such that $f(0)=0$ and *f* is right continuous on $(0,h)$. The (complex-valued) duality pairing between *X* and $X^{*}$ is given by the Riemann–Stieltjes integral, for $\varphi \in X$ and $f \in X^{*}$,

$$ \langle f, \varphi \rangle := \int _{0}^{h} \varphi (-\theta )\,df( \theta ). $$

Results on scalar functions of bounded variation and the corresponding Riemann–Stieltjes integral can be extended to *Y*-valued functions, see [59].

It is possible to find an explicit representation of the adjoint operator $A_{0}^{*}$ and its corresponding domain $D(A_{0}^{*})$. The adjoint operator exists and is unique as the domain $D(A_{0})$ is dense.

#### Theorem 2

*The domain of* $A_{0}^{*}$ *is given by*

8 $$\begin{aligned} D\bigl(A_{0}^{*}\bigr) :=& \biggl\{ f\in \operatorname{NBV}\bigl([0,h];Y^{*}\bigr)| \textit{there exists } y^{*}\in D\bigl(B^{*}\bigr) \textit{ and } g\in \operatorname{NBV}\bigl([0,h];Y^{*} \bigr) \\ &{}\textit{with } g(h)=0 \textit{ such that } f(t) = y^{*} \chi _{0}(t) + \int _{0}^{t} g(\theta )\,d\theta \biggr\} \end{aligned}$$

*and the action of* $A_{0}^{*}$ *is given by* $A_{0}^{*} f = B^{*}y^{*}\chi _{0}+g$, *where* $\chi_{0}=1_{\left(0,h\right]}$, *i*.*e*. *the characteristic function of* $(0,h]$.

#### Proof

We first prove the inclusion ⊆ for the domain $D(A_{0}^{*})$. Let $f\in D(A_{0}^{*})$ and $\varphi \in D(A_{0})$. Without loss of generality we can write $A_{0}^{*} f = c\chi _{0} + g$, where $c \in Y^{*}$ and $g \in \operatorname{NBV}([0,h];Y^{*})$ and $g(h)=0$. Using the integration by parts formulas for Riemann–Stieltjes integrals [60, Appendix H], we obtain

9 $$ \begin{aligned} \int _{0}^{h} \dot{\varphi }(-\theta )\,df( \theta ) &= \langle f, A_{0} \varphi \rangle \\ & = \bigl\langle A_{0}^{*} f, \varphi \bigr\rangle \\ & = \langle c\chi _{0} + g, \varphi \rangle \\ &= \int _{0}^{h} \varphi (-\theta )\,d\bigl(c\chi _{0}(\theta )\bigr) + \int _{0}^{h} \varphi (-\theta )\,dg(\theta ) \\ &= \bigl\langle c, \varphi (0) \bigr\rangle + \bigl\langle g(\theta ), \varphi (- \theta ) \bigr\rangle |_{0}^{h} - \int _{0}^{h} g(\theta )\,d\varphi (- \theta ) \\ &= \bigl\langle c, \varphi (0) \bigr\rangle + \int _{0}^{h} \bigl\langle g(\theta ), \dot{ \varphi }(-\theta ) \bigr\rangle \,d\theta . \end{aligned} $$

We will now want to use some limiting argument. However, the Riemann–Stieltjes integral lacks good convergence properties. In the scalar case, we could interpret this integral as a Lebesque–Stieltjes integral, which has better convergence properties. For a general Banach space *Y* and continuous integrands, the equivalent would be the Bartle integral [61, 62]. The Bartle integral has an equivalent theorem to the Lebesque dominated convergence theorem. For uniformly bounded, pointwise converging sequences, we can interchange the limit and the integral [62, Theorem 6].

For some $0< s< t\leq h$ and $y\in Y$, we may choose $(\dot{\varphi }_{n})_{n\in \mathbb{N}}$ as a uniformly bounded sequence in *X* such that $\dot{\varphi _{n}}(0)=\varphi _{n}(0)=0$ and it converges pointwise to $y1_{\left[-t,-s\right]}$, i.e. the characteristic function of $[-t,-s]$. We then substitute *φ* for $\varphi _{n}$ in (9)

$$ \int _{0}^{h} \dot{\varphi }_{n}(- \theta )\,df(\theta ) = \int _{0}^{h} \bigl\langle g(\theta ), \dot{ \varphi }_{n}(-\theta ) \bigr\rangle \,d\theta . $$

Taking the limit as $n \rightarrow \infty $, using the dominated convergence of the Bartle integral, we get that

$$\begin{array}{rl} & \int_{0}^{h}y1_{\left[-t,-s\right]}(-\theta )\;df(\theta )=\int_{0}^{h}\langle g(\theta ),y1_{\left[-t,-s\right]}(-\theta )\rangle \;d\theta ,\\ & \langle f(t)-f(s),y\rangle =\int_{s}^{t}\langle g(\theta ),y\rangle \;d\theta . \end{array}$$

Since *y* was arbitrary, we infer that

$$ f(t)= f(s) + \int _{s}^{t} g(\theta )\,d\theta . $$

Letting $s\downarrow 0$, we obtain for $t\in [0,h]$

$$ f(t)= y^{*}\chi _{0}(t) + \int _{0}^{t} g(\theta )\,d\theta , $$

where $y^{*} = \lim_{s\downarrow 0} f(s)$. Now we substitute this formula for *f* into $\langle f, A_{0} \varphi \rangle $ and use integration by parts and the fact that $\dot{\varphi }(0)=B\varphi (0)$ to find that

$$\begin{aligned} \langle f, A_{0} \varphi \rangle &= \bigl\langle y^{*} , \dot{\varphi }(0) \bigr\rangle + \int _{0}^{h} \bigl\langle g(\theta ), \dot{ \varphi }(-\theta ) \bigr\rangle \,d\theta \\ &= \bigl\langle y^{*} , B\varphi (0) \bigr\rangle + \int _{0}^{h} \bigl\langle g( \theta ), \dot{ \varphi }(-\theta ) \bigr\rangle \,d\theta . \end{aligned}$$

We compare this to equation (9)

$$ \langle f, A_{0} \varphi \rangle = \bigl\langle c, \varphi (0) \bigr\rangle + \int _{0}^{h} \bigl\langle g(\theta ), \dot{ \varphi }(-\theta ) \bigr\rangle \,d \theta . $$

Since $\varphi (0)$ can be chosen arbitrary, $\langle y^{*} , B\varphi (0) \rangle = \langle c, \varphi (0) \rangle $ implies that $c \in D(B^{*})$ and $c=B^{*} y^{*}$.

Finally we prove the other inclusion ⊇ for the domain $D(A_{0}^{*})$ and simultaneously obtain the formula for the action of $A_{0}^{*}$. Let *f* be of the form in (8), then by the above computations we find that

$$ \langle f, A_{0} \varphi \rangle = \bigl\langle B^{*}y^{*} , \varphi (0) \bigr\rangle + \int _{0}^{h} \varphi (-\theta )\,dg(\theta ) = \bigl\langle B^{*}y^{*} \chi _{0}+g, \varphi \bigr\rangle . $$

 □

We can characterise the sun-dual $X^{\odot }$ as the subspace of $X^{*}$, where $T_{0}^{*}$ is strongly continuous, or equivalently $X^{\odot } = \overline{D(A_{0}^{*})}$, where the closure is with respect to the norm on $X^{*}$. Similarly we can characterise the sun-dual $Y^{\odot }$ as the subspace of $Y^{*}$, where $B^{*}$ is strongly continuous, or equivalently $Y^{\odot } = \overline{D(B^{*})}$, where the closure is with respect to the norm on $Y^{*}$. In case *B* is the diffusion operator, see A for an explicit characterisation of $Y^{\odot }$.

The following theorem can be proved by showing that $T_{0}^{*}$ is strongly continuous on some set *E* given by (10), that $D(A_{0}^{*})\subseteq E$, and that *E* is closed.

#### Theorem 3

([5, Theorem 1 and Remark 4])

*The space* $X^{\odot }$, *the sun*-*dual of* *X* *with respect to* $T_{0}$, *is given by the set*

10 $$ \begin{aligned} &\biggl\{ f:[0,h]\rightarrow Y^{*}| \textit{ there exists } y^{\odot }\in Y^{\odot }\textit{ and } g\in L^{1}\bigl([0,h];Y^{*}\bigr) \\ &\quad \textit{such that } f(t) = y^{\odot }\chi _{0}(t) + \int _{0}^{t} g( \theta )\,d\theta \biggr\} . \end{aligned} $$

*Furthermore*, *the map* $\iota : Y^{\odot }\times L^{1}([0,h];Y^{*})\rightarrow X^{\odot }$ *defined by*

11 $$ \iota \bigl(y^{\odot }, g\bigr) (t) := y^{\odot } \chi _{0}(t) + \int _{0}^{t} g( \theta )\,d\theta \quad \forall t\in [0,h] $$

*is an isometric isomorphism*.

From now on we identify $X^{\odot }$ with $Y^{\odot }\times L^{1} ([0,h];Y^{*})$. The corresponding duality pairing between *X* and $X^{\odot }$ is then given by

12 $$ \bigl\langle \varphi ^{\odot }, \varphi \bigr\rangle := \bigl\langle y^{\odot }, \varphi (0) \bigr\rangle + \int _{0}^{h} \bigl\langle g(\theta ), \varphi (- \theta ) \bigr\rangle \,d\theta . $$

Now we can describe the action of $T_{0}^{\odot }$ and $A_{0}^{\odot }$, the restrictions of the operators $T_{0}^{*}$ and $A_{0}^{*}$ to the subspace $X^{\odot }$.

#### Definition 4

The strongly continuous semigroup $T_{1}$ on $L^{1}([0,h];Y^{*})$ is defined as

13 $$ \bigl(T_{1}(t)g\bigr) (\theta ) := \textstyle\begin{cases} g(t+\theta ) &t+\theta \in [0,h], \\ 0 &t+\theta > h. \end{cases} $$

#### Theorem 5

([5, Theorem 1])

*For the action of* $T_{0}^{\odot }$ *on* $X^{\odot }$, *we have*

14 $$ T_{0}^{\odot }(t) \bigl(y^{\odot },g\bigr) := \biggl(S^{\odot }(t) y^{\odot }+ \int _{0}^{ \min (t,h)} S^{*}(t-\theta )g( \theta )\,d\theta , T_{1}(t)g \biggr), $$

*where the integral is the weak*^∗^ *Lebesque integral with values in* $Y^{\odot }$.

#### Theorem 6

*For the sun*-*dual of* $A_{0}$ *on* $X^{\odot }$, *we have that*

15 $$ \begin{aligned} D\bigl(A_{0}^{\odot } \bigr)= {}&\bigl\{ \bigl(y^{\odot },g\bigr) | g \in \operatorname{AC} \bigl([0,h];Y^{*}\bigr) \textit{ with } g(h)=0, y^{\odot }\in D\bigl(B^{*}\bigr)\\ &{}\textit{and } B^{*} y^{\odot }+ g(0)\in Y^{\odot } \bigr\} \end{aligned} $$

*and* $A_{0}^{\odot }(y^{\odot },g) = (B^{*} y^{\odot }+ g(0),\dot{g})$, *with* *ġ* *a function in* $L^{1}([0,h];Y^{*})$ *such that*

16 $$ g(t) = g(0) + \int _{0}^{t} \dot{g}(\theta )\,d\theta $$

*for* $t \in [0,h]$.

#### Proof

By definition

$$ D\bigl(A_{0}^{\odot }\bigr) := \bigl\{ \varphi ^{\odot }\in X^{\odot }| \iota \varphi ^{\odot }\in D \bigl(A_{0}^{*}\bigr), A_{0}^{*} \iota \varphi ^{\odot }\in \iota \bigl(X^{\odot }\bigr) \bigr\} $$

and $\iota A_{0}^{\odot }\varphi ^{\odot }= A_{0}^{*} \iota \varphi ^{\odot }$. We first prove the equivalence of the definition and (15).

Let $\varphi ^{\odot }=(y^{\odot },g) \in X^{\odot }$ such that $\iota \varphi ^{\odot }\in D(A_{0}^{*})$ and $A_{0}^{*} \iota \varphi ^{\odot }\in \iota (X^{\odot })$. Recall that the embedding *ι* is given by (11)

$$ \iota \varphi ^{\odot }(t) = y^{\odot }\chi _{0}(t) + \int _{0}^{t} g( \theta )\,d\theta . $$

From Theorem 2, we can conclude that $\iota \varphi ^{\odot }\in D(A_{0}^{*})$ implies that $y^{\odot }\in D(B^{*})$ and $g\in \operatorname{NBV}([0,h];Y^{*})$ with $g(h)=0$. As $A_{0}^{*} \iota \varphi ^{\odot }= B^{*} y^{\odot }\chi _{0}+g \in \iota (X^{\odot })$, Theorem 3 implies that $B^{*}y^{\odot }+g(0+)\in Y^{\odot }$, and we can write *g* as

$$ g(t) = g(0+)\chi _{0} + \int _{0}^{t} \dot{g}(\theta )\,d\theta , $$

where $g(0+)= \lim_{t \downarrow 0} g(t)$ and *ġ* some function in $L^{1}([0,h];Y^{\odot })$. Hence *g* is absolutely continuous on $(0,h]$. As *g* is an $L^{1}$-function (class), we may redefine $g(0):=g(0+)$ to get an absolutely continuous function on $[0,h]$.

Conversely, let $\varphi ^{\odot }=(y^{\odot },g) \in X^{\odot }$ such that it is in the right-hand side of (15). From Theorem 2 and the fact that $g-g(0)\in \operatorname{NBV}([0,h];Y^{*})$, we conclude that $\iota \varphi ^{\odot }\in D(A_{0}^{*})$ and that $A_{0}^{*} \iota \varphi ^{\odot }= (B^{*} y^{\odot }+ g(0))\chi _{0} + g$. As *g* is absolutely continuous and $B^{*} y^{\odot }+ g(0)\in Y^{\odot }$, this implies that $A_{0}^{*} \iota \varphi ^{\odot }= \iota (B^{*} y^{\odot }+ g(0),\dot{g}) \in \iota (X^{\odot })$. Hence, $A_{0}^{\odot }\varphi ^{\odot }= (B^{*} y^{\odot }+ g(0),\dot{g})$. □

### Characterisation of the sun-star space

We can represent $X^{\odot *}$, the dual of $X^{\odot }$, as $Y^{\odot *} \times (L^{1}([0,h];Y^{*})^{*}$, where $Y^{\odot *}$ is the dual of $Y^{\odot }$. In case *B* is the diffusion operator, $Y^{\odot *}$ is explicitly characterised in Appendix A.

In general, $(L^{1}([0,h];Y^{*})^{*}$ cannot be identified with $L^{\infty }([-h,0];Y^{**})$. However, the latter space can be embedded into the former.

#### Theorem 7

([63, Remark 1.4.18, Theorem 1.4.19])

*There exists an isometric embedding of* $L^{\infty }([-h,0];Y^{**})$ *into* $(L^{1}([0,h];Y^{*})^{*}$ *with the duality pairing*

$$ \langle \varphi ,g \rangle = \int _{0}^{h} \bigl\langle \varphi (-\theta ),g( \theta ) \bigr\rangle \,d\theta $$

*for* $g\in L^{1}([0,h];Y^{*})$ *and* $\varphi \in L^{\infty }([-h,0];Y^{**})$.

*Moreover*, $(L^{1}([0,h];Y^{*})^{*}$ *can be identified with* $L^{\infty }([-h,0];Y^{**})$ *if and only if* $Y^{**}$ *has the Radon–Nikodym property*.

#### Lemma 8

(Dunford–Pettis)

*If* *Y* *is reflexive*, *then it has the Radon–Nikodym property*.

We can embed both *Y* and *X* into $Y \times X$ which is a subspace of $Y^{\odot *} \times L^{\infty }([-h,0];Y^{**})$. The canonical embedding $j: X \rightarrow X^{\odot *}$ is defined as $\langle j \varphi ,\varphi ^{\odot }\rangle = \langle \varphi ^{\odot }, \varphi \rangle $. The continuous embedding $\ell : Y \rightarrow X^{\odot *}$ is defined as $\ell = (j_{Y} y, 0)$, where $j_{Y}$ is the canonical embedding of *Y* into $Y^{\odot *}$. [5] It is possible to find an explicit representation of *j*.

#### Lemma 9

*For* $\varphi \in X$, $j\varphi = (j_{Y} \varphi (0),\varphi )$. *Moreover*, *j* *is a continuous embedding and* $j^{-1}: j(X) \rightarrow X$ *is bounded*. $T_{0}^{\odot *}(t)j = j T_{0}(t)$, *consequently* $j(X)$ *is contained in* $X^{\odot \odot }$, *which is the subspace of* $X^{\odot *}$ *on which* $T_{0}^{\odot *}$ *is strongly continuous*.

#### Proof

Let $\varphi \in X$ and $\varphi ^{\odot }=(y^{\odot },g) \in X^{\odot }$, then

$$\begin{aligned} \bigl\langle j \varphi ,\varphi ^{\odot }\bigr\rangle &= \bigl\langle \varphi ^{\odot }, \varphi \bigr\rangle \\ &= \bigl\langle y^{\odot }, \varphi (0) \bigr\rangle + \int _{0}^{h} \bigl\langle g( \theta ), \varphi (-\theta ) \bigr\rangle \,d\theta \\ &= \bigl\langle j_{Y} \varphi (0), y^{\odot }\bigr\rangle + \int _{0}^{h} \bigl\langle \varphi (-\theta ), g(\theta ) \bigr\rangle \,d\theta \\ &= \bigl\langle \bigl(j_{Y}\varphi (0),\varphi \bigr), \varphi ^{\odot }\bigr\rangle . \end{aligned}$$

Hence $j\varphi = (j_{Y} \varphi (0),\varphi )$. The other statements are generally known to hold for the canonical embedding of *X* into $X^{\odot *}$ [39, Appendix II, Cor. 3.16, Prop. 3.17]. □

As we do not have an explicit norm or measure on $(L^{1}([0,h];Y^{*})^{*}$, we cannot say anything in general about $A_{0}^{\odot *}$. However, it is possible to find a representation of $A_{0}^{\odot *}$ restricted to the space ${Y^{\odot *} \times L^{\infty }([-h,0];Y^{**})}$.

#### Theorem 10

*For* $(y^{\odot *},\varphi ) \in X^{\odot *}$, *the following statements are equivalent*:

1. $(y^{\odot *},\varphi ) \in D(A_{0}^{\odot *})$ *and* $A_{0}^{\odot *}(y^{\odot *},\varphi )\in Y^{\odot *} \times L^{\infty }([-h,0];Y^{**})$;
2. *φ* *has an a*.*e*. *derivative* $\dot{\varphi } \in L^{\infty }([-h,0];Y^{**})$ *for which*
  $$ \varphi (t) = y^{\odot *} - \int _{t}^{0} \dot{\varphi }(\theta )\,d\theta $$
  *and* $\varphi (0)=y^{\odot *}\in D(B^{\odot *})$.

*In this case the action of* $A_{0}^{\odot *}$ *is given by* $A_{0}^{\odot *}(y^{\odot *},\varphi ) = (B^{\odot *} y^{\odot *}, \dot{\varphi })$.

#### Proof

Let $(y^{\odot *},\varphi ) \in D(A_{0}^{\odot *})$ such that $A_{0}^{\odot *}(y^{\odot *},\varphi )= (\gamma , \psi )\in Y^{\odot *} \times L^{\infty }([-h,0];Y^{**})$, and let $(y^{\odot },g) \in D(A_{0}^{\odot })$. We have that

17 $$ \begin{aligned} \bigl\langle y^{\odot *}, B^{*} y^{\odot }+g(0) \bigr\rangle + \langle \varphi , \dot{g} \rangle &= \bigl\langle \bigl(y^{\odot *},\varphi \bigr), A_{0}^{ \odot }\bigl(y^{\odot },g\bigr) \bigr\rangle \\ &= \bigl\langle A_{0}^{\odot *}\bigl(y^{\odot *}, \varphi \bigr),\bigl(y^{\odot },g\bigr) \bigr\rangle \\ &= \bigl\langle \gamma , y^{\odot }\bigr\rangle + \int _{0}^{h} \bigl\langle \psi (- \theta ),g( \theta ) \bigr\rangle \,d\theta . \end{aligned} $$

Let $\Phi \in L^{\infty }([-h,0];Y^{**})$ such that

$$ \Phi (t) = \Phi (0) - \int _{t}^{0} \psi (\theta )\,d\theta . $$

Then, by Lemma 46 and Theorem 6, i.e. $g(h)=0$, we can rewrite (17) as

18 $$ \bigl\langle y^{\odot *}, B^{*} y^{\odot }+g(0) \bigr\rangle = \bigl\langle \gamma , y^{\odot } \bigr\rangle + \bigl\langle \Phi (0), g(0) \bigr\rangle + \langle \Phi - \varphi , \dot{g} \rangle . $$

Taking $g\equiv 0$, we get that $\langle y^{\odot *}, B^{*} y^{\odot }\rangle = \langle \gamma , y^{\odot }\rangle $ for all $y^{\odot }\in Y^{\odot }$ such that $B^{*} y^{\odot }\in Y^{\odot }$ by Theorem 6. Hence $y^{\odot }\in D(B^{\odot })$, which implies that $y^{\odot *} \in D(B^{\odot *})\subseteq Y^{\odot \odot }$ and $\gamma = B^{\odot *}y^{\odot *}$. As $Y^{\odot \odot }$ can be embedded in $Y^{**}$ [64, Corollary 4.2], we find that $y^{\odot *} \in Y^{**}$. Furthermore, by [64, Theorem 4.3] we have, for all $y^{\odot *} \in D(B^{\odot *})$ and $y^{\odot }\in D(B^{*})$,

$$ \bigl\langle B^{\odot *} y^{\odot *}, y^{\odot }\bigr\rangle = \bigl\langle y^{\odot *}, B^{*} y^{\odot }\bigr\rangle . $$

Alternatively, we take $\Phi (0) = y^{\odot *}$, $g(0) = -\int _{0}^{h} \dot{g}(\theta )\,d\theta $ and $y^{\odot }\in D(B^{*})$ such that $B^{*}y^{\odot }+ g(0) \in Y^{\odot }$. Then (18) reduces to $\langle \Phi -\varphi , \dot{g} \rangle =0$ for all $\dot{g}\in L^{1}([0,h];Y^{*})$, hence $\Phi \equiv \varphi $.

Conversely, let $(y^{\odot *},\varphi ) \in Y^{\odot *} \times L^{\infty }([-h,0];Y^{**})$, where $\varphi (0)=y^{\odot *}\in D(B^{\odot *})$ and *φ* has an a.e. derivative $\dot{\varphi } \in L^{\infty }([-h,0];Y^{**})$ for which

$$ \varphi (t) = y^{\odot *} - \int _{t}^{0} \dot{\varphi }(\theta )\,d\theta . $$

Then again using Lemma 46 we get that, for any $(y^{\odot },g) \in D(A_{0}^{\odot })$,

$$\begin{aligned} \bigl\langle \bigl(y^{\odot *},\varphi \bigr), A_{0}^{\odot } \bigl(y^{\odot },g\bigr) \bigr\rangle &= \bigl\langle y^{\odot *}, B^{*} y^{\odot }+g(0) \bigr\rangle + \int _{0}^{h} \bigl\langle \varphi (-\theta ), \dot{g}(\theta ) \bigr\rangle \,d\theta \\ &= \bigl\langle y^{\odot *}, B^{*} y^{\odot }\bigr\rangle + \int _{0}^{h} \bigl\langle \dot{\varphi }(- \theta ),g(\theta ) \bigr\rangle \,d\theta \\ &= \bigl\langle B^{\odot *}y^{\odot *}, y^{\odot }\bigr\rangle + \int _{0}^{h} \bigl\langle \dot{\varphi }(- \theta ),g(\theta ) \bigr\rangle \,d\theta \\ &= \bigl\langle \bigl(B^{\odot *} y^{\odot *}, \dot{\varphi }\bigr), \bigl(y^{\odot }, g\bigr) \bigr\rangle . \end{aligned}$$

Hence $A_{0}^{\odot *}(y^{\odot *},\varphi ) = (B^{\odot *} y^{\odot *}, \dot{\varphi }) \in Y^{\odot *} \times L^{\infty }([-h,0];Y^{**})$. □

#### Corollary 11

*For* $\varphi \in X$, *the following statements are equivalent*:

1. $j\varphi \in D(A_{0}^{\odot *})$ *and* $A_{0}^{\odot *}j\varphi \in Y^{\odot *} \times L^{\infty }([-h,0];Y^{**})$;
2. $j_{Y}\varphi (0) \in D(B^{\odot *})$ *and* *φ* *has an a*.*e*. *derivative* $\dot{\varphi } \in L^{\infty }([-h,0];Y)$.

*In this case*, *the action of* $A_{0}^{\odot *}$ *is given by* $A_{0}^{\odot *}j\varphi = (B^{\odot *} j_{Y} \varphi (0), \dot{\varphi })$.

#### Proof

This follows immediately from Theorem 10 and Lemma 9. □

Note that, for $A_{0}^{\odot *}$, the rule for extension $\dot{\varphi }(0) = B\varphi (0)$ is no longer included in the domain of $A_{0}^{\odot *}$, but is represented in the action of $A_{0}^{\odot *}$, which resolves the problem with $A_{0}$ stated at the beginning of this section.

The previous theorem allows us to formulate an equivalence between the sun-reflexivity of *X*, i.e. $X^{\odot \odot } = j(X)$ and the ordinary reflexivity of *Y*, i.e. $Y^{**} = j_{Y}(Y)$

#### Theorem 12

*X* *is sun*-*reflexive with respect to* $T_{0}$ *if and only if* *Y* *is reflexive*.

#### Proof

Suppose that *Y* is reflexive. Then, by Theorem 7 and Lemma 8, $X^{\odot *}$ can be represented as $Y^{\odot *} \times L^{\infty }([-h,0];Y)$ and hence the full domain of $A_{0}^{\odot *}$ is given by Theorem 10:

$$ D\bigl(A_{0}^{\odot *}\bigr)= \bigl\{ \bigl(y^{\odot *}, \varphi \bigr)\in X^{\odot *} | \varphi (0)=y^{\odot *}\in D \bigl(B^{\odot *}\bigr), \varphi \text{ has an a.e. derivative} \bigr\} . $$

We use that $X^{\odot \odot }$ is the closure of $D(A_{0}^{\odot *})$ with respect to the norm on $X^{\odot *}$. First the closure of $D(B^{\odot *})$ with respect to the $Y^{\odot *}$-norm results in the space $Y^{\odot \odot }$. As reflexivity implies sun-reflexivity [65, Corollary 2.5], we have that $Y^{\odot \odot }= j_{Y}(Y)$. Next we note that $C^{1}$ functions are dense in the continuous functions and $C^{0}$ is closed with respect to the $L^{\infty }$-norm. Hence we conclude that

$$ X^{\odot \odot } = \bigl\{ \bigl(y^{\odot \odot },\varphi \bigr)\in j_{Y}(Y) \times C\bigl([-h,0];Y\bigr)| \varphi (0)=y^{\odot \odot } \bigr\} = j(X). $$

Conversely, suppose that *Y* is not reflexive. From Theorem 7, $Y^{\odot *} \times L^{\infty }([-h,0];Y)$ is a subset of $X^{\odot *}$ and hence

$$ \bigl\{ \bigl(y^{\odot *},\varphi \bigr)\in X^{\odot *} | \varphi (0)=y^{\odot *}\in D\bigl(B^{ \odot *}\bigr), \varphi \text{ has an a.e. derivative}\bigr\} \subseteq D\bigl(A_{0}^{ \odot *} \bigr). $$

Taking the norm closure of both sides, we conclude that

$$ \bigl\{ \bigl(y^{\odot \odot },\varphi \bigr)\in Y^{\odot \odot } \times C \bigl([-h,0];Y^{**}\bigr)| \varphi (0)=y^{\odot \odot }\bigr\} \subseteq X^{\odot \odot }. $$

As *Y* is not reflexive, $C([-h,0];Y)$ is a proper subset of $C([-h,0];Y^{**})$. Hence $j(X)$ is a proper subset of $X^{\odot \odot }$, so *X* is not sun-reflexive. □

In case *B* is the diffusion operator, we use that *Y* is the space of continuous functions. As this is a non-reflexive Banach space, *X* in this case is not sun-reflective.

### Variation-of-constants formulation

As the space $X^{\odot *}$ solves the problems mentioned at the beginning of this section, we can formulate a variation-of-constants formula for (ADDE) as an abstract integral equation

AIE $$ u_{t}=T_{0}(t)\varphi +j^{-1} \int _{0}^{t} T_{0}^{\odot *}(t- \tau ) \ell G(u_{\tau })\,d\tau . $$

Here the embeddings *j* and *ℓ* are as defined before Lemma 9. As the integrand of (AIE) takes values in $X^{\odot *}$, the integral is taken to be a weak^∗^ integral. It is possible to show that the integral maps to the range of $j(X)$ and hence (AIE) is well defined.

#### Lemma 13

([5, Proposition 8])

*Let* $u \in C(\mathbb{R}^{+},Y)$ *be given*, *then*

19 $$ \int _{0}^{t} T_{0}^{\odot \ast } (t-\tau ) \ell u(\tau )\,d\tau = j \psi \quad \forall t\geq 0, $$

*where*

20 $$ \psi (\theta ):= \int _{0}^{\max \{(t+\theta ), 0\}}S(t-\tau +\theta )u( \tau )\,d\tau \quad \forall \theta \in [-h,0]. $$

*Moreover*,

21 $$ \Vert \psi \Vert \leq M e^{\omega h} \frac{e^{\omega t}-1}{\omega }\sup_{0 \leq \tau \leq t} \bigl\Vert u(\tau ) \bigr\Vert \quad \forall t\geq 0, $$

*where* $M,\omega >0$ *are such that* $\|S(t)\| \leq M e^{\omega t}$ *for all* $t\geq 0$.

The Banach fixed point theorem in combination with the bound in (21) gives the existence of a unique global solution of (AIE).

#### Corollary 14

([5, Corollary 9])

*Let* $G:X\rightarrow Y$ *be globally Lipschitz continuous*. *For every initial condition* $\varphi \in X$, *there exists a unique solution* $v\in C(\mathbb{R}_{+},X)$ *such that* $u_{t}=v(t)$ *satisfies* (AIE) *for all* $t \geq 0$.

We would like to show that this unique solution of (AIE) can be translated over to a (classical) solution of (ADDE). However, this is in general not the case when *B* is unbounded. Therefore we recall a weaker solution concept from [44].

#### Definition 15

A function $u\in C([-h,\infty );Y)$ is called a *classical solution* of (ADDE) if *u* is continuously differentiable on $\mathbb{R}_{+}$, $u(t)\in D(B)$ for all $t\geq 0$ and *u* satisfies (ADDE).

#### Definition 16

A function $u\in C([-h,\infty );Y)$ is called a *mild solution* of (ADDE) if $u_{0} =\varphi $ and *u* satisfies

22 $$ u(t)=S(t)\varphi (0) + \int _{0}^{t} S(t-\tau ) G(u_{\tau })\,d\tau\quad \forall t\geq 0. $$

Note that Definition 15 is quite restrictive as only specific initial conditions $\varphi \in X$ are admissible. There is the following correspondence between classical and mild solutions of (ADDE)

#### Lemma 17

([44, Theorem 2.1.4])

*A classical solution of* (ADDE) *is also a mild solution of* (ADDE)

*Conversely*, *when* *G* *has a globally Lipschitz continuous Fréchet derivative and* $\varphi \in C^{1}([-h,0];Y)$, $\varphi (0) \in D(B)$ *and* $\dot{\varphi }(0) = B\varphi (0) + G(\varphi )$, *then a mild solution of* (ADDE) *is also a classical solution of* (ADDE).

Note that Theorem 25 implies that the conditions in the second statement, starting with conversely, are equivalent to the condition that $\varphi \in D(A)$.

It is possible to construct a one-to-one correspondence between solutions of (AIE) and mild solutions of (ADDE).

#### Theorem 18

([5, Theorem 16])

*Let* $\varphi \in X$ *be an initial condition*. *The following two statements hold*.

1. *Suppose that* *u* *is a mild solution of* (ADDE). *Define* $v:\mathbb{R}_{+}\rightarrow X$ *by*
  $$ v(t):=u_{t} \quad \forall t\geq 0. $$
  *Then* *v* *is a solution of* (AIE).
2. *Suppose that* *v* *is a solution of* (AIE). *Define* $u:[-h,\infty )\rightarrow Y$ *by*
  $$ u(t):= \textstyle\begin{cases} \varphi (t) &-h\leq t\leq 0, \\ v(t)(0) & t\geq 0. \end{cases} $$
  *Then* *u* *is a mild solution of* (ADDE).

#### Corollary 19

*Suppose that* *G* *is a globally Lipschitz operator and it has a globally Lipschitz Fréchet derivative*, *then for all* $\varphi \in C^{1}([-h,0];Y)$ *with* $\varphi (0) \in D(B)$ *and* $\dot{\varphi }(0) = B\varphi (0) + G(\varphi )$, *there exists a unique classical solution of* (ADDE).

### Linearisation

We want to investigate the behaviour near a fixed point. We will show that for the linearised problem we can perturb the semigroup $T_{0}$ with generator $A_{0}$ to a semigroup *T* with generator *A*. In the next section we investigate the spectral properties of *A*.

Linearising equation (ADDE) near a fixed point *u*, which we take without loss of generality to be $u\equiv 0$, results in the linear problem (LINP).

LINP $$ \textstyle\begin{cases} \dot{u}(t)=Bu(t)+DG(0)u_{t}, \\ u_{0}=\varphi \in X. \end{cases} $$

As with the general nonlinear problem, we can define an abstract integral equation

AIE $$ u_{t} = T_{0}(t) \varphi + j^{-1} \int _{0}^{t} T_{0}^{\odot *}(t-s)L u_{t}\,ds, $$

where $L := \ell DG(0)$. Then, due to Lemma 13 and Corollary 14, we can define the strongly continuous semigroup $T(t)\varphi :=u_{t}$ when $DG(0)$ is globally Lipschitz.

#### Lemma 20

([5, Theorem 19])

*Let* $DG(0)$ *be globally Lipschitz continuous*, *then there exists a unique strongly continuous semigroup* *T* *on* *X* *such that*

23 $$ T(t)\varphi = T_{0}(t) \varphi + j^{-1} \int _{0}^{t} T_{0}^{\odot \ast } LT(\tau ) \varphi \,d\tau $$

*for all* $\varphi \in X$ *and for all* $t\geq 0$.

The strongly continuous semigroup *T* has a generator *A*. We want to establish how the perturbed generator *A* relates to the original generator $A_{0}$, which can be done using the sun-star framework. A technical detail which we need to check is that the sun-dual space $X^{\odot }$ is the same with respect to *T* and $T_{0}$.

#### Lemma 21

([5, Proposition 20])

$X^{\odot }$ *is also the maximal subspace of strong continuity of the adjoint semigroup* $T^{*}$ *on* $X^{*}$. *The adjoint generator* $A^{*}$ *is given by*

24 $$ A^{*}=A_{0}^{*} + L^{*} \quad \textit{with } D\bigl(A^{*}\bigr)=D\bigl(A_{0}^{*} \bigr) $$

*and the generator* $A^{\odot }$ *of the* $T^{\odot }$ *is given by*

25 $$ A^{\odot }=A_{0}^{\odot }+ L^{\odot }\quad \textit{with } D\bigl(A^{\odot }\bigr)=D\bigl(A_{0}^{\odot }\bigr). $$

*Finally*, $X^{\odot \odot }$ *is also the maximal subspace of strong continuity of the sun*-*star semigroup* $T^{\odot \odot }$.

One could think that we could extend this argument and show that $D(A^{\odot \ast })=D(A_{0}^{\odot \ast })$ and $A^{\odot \ast }=A_{0}^{\odot \ast }+L j^{-1}$. However, this is not the case when we lack sun-reflexivity, i.e. $X^{\odot \odot }\neq j(X)$. We can circumvent these problems by restricting the domain to $j(X)$.

#### Lemma 22

([5, Proposition 22])

*It holds that*

26 $$ D\bigl(A^{\odot \ast }\bigr)\cap j (X) = D \bigl(A_{0}^{\odot \ast }\bigr) \cap j (X) $$

*and* $A^{\odot \ast }=A_{0}^{\odot \ast } + L j^{-1}$ *on this subspace*.

We can extend Corollary 11 for $A_{0}^{\odot *}$ to $A^{\odot *}$, which will be needed for the computation of normal form coefficients.

#### Corollary 23

*For* $\varphi \in X$, *the following statements are equivalent*:

1. $j\varphi \in D(A^{\odot *})$ *and* $A^{\odot *}j\varphi \in Y^{\odot *} \times L^{\infty }([-h,0];Y^{**})$;
2. $j_{Y}\varphi (0) \in D(B^{\odot *})$ *and* *φ* *has an a*.*e*. *derivative* $\dot{\varphi } \in L^{\infty }([-h,0];Y)$.

*In this case*, *the action of* $A^{\odot *}$ *is given by* $A^{\odot *}j\varphi = (B^{\odot *} j_{Y} \varphi (0) + j_{Y} DG(0) \varphi , \dot{\varphi })$.

#### Proof

The statement on the domain follows immediately from Lemma 22 and Corollary 11. Furthermore, we have that

$$ A^{\odot *}j \varphi = A_{0}^{\odot *} j \varphi + \ell DG(0) \varphi = \bigl(B^{\odot *} j_{Y} \varphi (0), \dot{ \varphi }\bigr) + \bigl(j_{Y} DG(0) \varphi ,0\bigr). $$

 □

We are now able to state the result which relates *A* to $A_{0}$.

#### Theorem 24

([5, Corollary 23])

*For the generator* *A* *of the semigroup* *T*, *we have that*

27 $$ \begin{aligned} &D(A)=\bigl\{ \varphi \in X | j\varphi \in D\bigl(A_{0}^{\odot *}\bigr), A_{0}^{ \odot *}j \varphi + L \varphi \in j(X)\bigr\} , \\ &A= j^{-1}\bigl(A_{0}^{\odot *} j + L\bigr). \end{aligned} $$

We can cast (27) in a form which can also be found in Engel and Nagel [58, Theorem VI.6.1] by using Corollary 11.

#### Theorem 25

*For the generator* *A* *of the semigroup* *T*, *we have that*

28 $$ \begin{aligned} &D(A)=\bigl\{ \varphi \in C^{1}\bigl([-h,0];Y\bigr) | \varphi (0)\in D(B), \dot{\varphi }(0) = B\varphi (0) + DG(0)\varphi \bigr\} , \\ &A\varphi= \dot{\varphi }. \end{aligned} $$

#### Proof

Let $j\varphi \in D(A_{0}^{\odot *})$ and $A_{0}^{\odot *}j\varphi + L \varphi \in j(X)$. As $L\varphi \in j_{Y}(Y)\times \{0\}$, we have that $A_{0}^{\odot *}j\varphi \in Y^{\odot *} \times L^{\infty }([-h,0];Y^{**})$. By Corollary 11, $j_{Y}\varphi (0) \in D(B^{\odot *})$ and *φ* has an a.e. derivative $\dot{\varphi } \in L^{\infty }([-h,0];Y)$. Furthermore, we have that

$$ A_{0}^{\odot *}j\varphi + L \varphi = \bigl(B^{\odot *}j_{Y} \varphi (0) + j_{Y} DG(0)\varphi , \dot{\varphi }\bigr) \in j(X). $$

By Lemma 9 this implies that $B^{\odot *}j_{Y}\varphi (0) + j_{Y} DG(0)\varphi \in j_{Y}(Y)$, $\dot{\varphi } \in C([-h,0];Y)$ and $\dot{\varphi }(0) = B\varphi (0) + DG(0)\varphi $. Hence $\varphi \in C^{1}([-h,0];Y)$ and $B^{\odot *}j_{Y}\varphi (0) \in j_{Y}(Y)$.

Let $B^{\odot *}j_{Y}\varphi (0)=j_{Y} y$ with $y\in Y$. As $B^{\odot *}j_{Y}\varphi (0) \in Y^{\odot \odot }$, $j_{Y}\varphi (0)\in D(B^{\odot \odot })$. Let $S^{\odot \odot }$ be the strongly continuous semigroup generated by $B^{\odot \odot }$. This implies that

$$ j_{Y}\frac{1}{t}\bigl(S(t)\varphi (0)-\varphi (0)\bigr)= \frac{1}{t}\bigl(S^{\odot \odot }(t)j_{Y}\varphi (0)-j_{Y}\varphi (0)\bigr) $$

for all $t>0$ [39, Appendix II Proposition 3.17]. By the continuity of $j_{Y}^{-1}$, this converges in norm as $t\downarrow 0$ to $j_{Y} B\varphi (0) = B^{\odot \odot } j_{Y}\varphi (0)$ with $\varphi (0)\in D(B)$.

Conversely, let $\varphi \in C^{1}([-h,0];Y)$, $\varphi (0)\in D(B)$ and $\dot{\varphi }(0) = B\varphi (0) + DG(0)\varphi $. Furthermore, let $y^{\odot }\in D(B^{\odot })$, then

$$ \bigl\langle j_{Y} B\varphi (0), y^{\odot }\bigr\rangle = \bigl\langle y^{\odot }, B \varphi (0) \bigr\rangle = \bigl\langle B^{\odot }y^{\odot }, \varphi (0) \bigr\rangle = \bigl\langle j_{Y} \varphi (0), B^{\odot }y^{\odot }\bigr\rangle . $$

Hence $j_{Y} \varphi (0)\in D(B^{\odot *})$ and, by Corollary 11, $j\varphi \in D(A_{0}^{\odot *})$. Furthermore,

$$\begin{aligned} A_{0}^{\odot *}j\varphi + L \varphi &=\bigl(B^{\odot *}j_{Y} \varphi (0) + j_{Y} DG(0)\varphi , \dot{\varphi }\bigr) \\ &= \bigl(j_{Y} B\varphi (0) + j_{Y} DG(0)\varphi , \dot{\varphi }\bigr) \\ & = j \dot{\varphi }\in j(X). \end{aligned}$$

Finally, for the action of *A*, we derive

$$ A\varphi = j^{-1}\bigl(A_{0}^{\odot *} j + L \bigr)\varphi = j^{-1}\bigl(j_{Y} B \varphi (0) + j_{Y} DG(0)\varphi , \dot{\varphi }\bigr) =\dot{\varphi }. $$

 □

### Spectral properties

In this section we state some results on the spectrum of the operator *A*, notably its essential spectrum and a method for computing its eigenvalues.

For an operator *A* on *X*, the resolvent set $\rho (A)$ is the set of all $z\in \mathbb{C}$ such that the operator $z-A$ has a bounded inverse. The resolvent operator $R(z,A): X\rightarrow D(A)$ is then defined as $R(z,A)= (z-A)^{-1}$ for $z \in \rho (A)$. The spectrum of *A*, $\sigma (A)=\mathbb{C}\setminus \rho (A)$ can be decomposed into the point spectrum $\sigma _{p}(A)$ and the essential spectrum $\sigma _{\mathrm{ess}}(A)$. We use Weyl’s definition of the essential spectrum, i.e. $\sigma _{\mathrm{ess}}(A):= \{\lambda \in \mathbb{C} | \lambda -A\mbox{ is not a Fredholm operator}\}$ [66]. Then $\sigma _{P}(A)=\sigma (A) \setminus \sigma _{\mathrm{ess}}(A)$ is the discrete spectrum, i.e. isolated eigenvalues with a finite dimensional eigenspace.

#### Lemma 26

*For the respective spectra*, *we have* $\sigma (A_{0})=\sigma (A_{0}^{*})=\sigma (A_{0}^{\odot })=\sigma (A_{0}^{ \odot *})=\sigma (B)$. *Furthermore*, $\sigma _{\mathrm{ess}}(A_{0})=\sigma _{\mathrm{ess}}(B)$.

#### Proof

We have that $\sigma (A_{0})=\sigma (A_{0}^{*})=\sigma (A_{0}^{\odot })=\sigma (A_{0}^{ \odot *})$ [58, Proposition IV.2.18].

Next we consider the eigenvalues of $A_{0}$. For some $\lambda \in \sigma (A_{0})$, we need to find $\varphi \in D(A_{0})$ such that $\dot{\varphi }=\lambda \varphi $. Clearly, this is the case if and only if $\varphi (\theta )= q e^{\lambda \theta }$ for $\theta \in [-h,0]$, with $q\in D(B)$ and $Bq = B \varphi (0) = \dot{\varphi }(0) = \lambda q$. Therefore $\lambda \in \sigma _{p}(A_{0})$ if and only if $\lambda \in \sigma _{p}(B)$ as the corresponding eigenspaces have the same dimension.

Finally, we show that $\rho (A_{0})=\rho (B)$, which completes the proof. If $z\in \rho (B)$, then we can find the resolvent of $A_{0}$ explicitly as for all $\varphi \in X$ and $\theta \in [-h,0]$, [58, Proposition VI.6.7]

29 $$ \bigl[R(z,A_{0}))\varphi \bigr](\theta )= e^{z \theta }R(z,B) \varphi (0) + \int _{ \theta }^{0} e^{z(\theta -s)}\varphi (s)\,ds. $$

Hence $z \in \rho (A_{0})$.

Conversely, suppose that $z \in \rho (A_{0})$, and let $y\in Y$. Then the constant function $\psi (\theta ) :=y$ for $\theta \in [-h,0]$ is in X and hence $\varphi := R(z,A_{0})\psi \in D(A_{0})$. This implies that $\varphi (0) \in D(B)$ and $(z-B)\varphi (0) = z \varphi (0) - \dot{\varphi }(0) = ((z-A_{0}) \varphi )(0) = \psi (0) = y$. Hence $z-B$ is surjective. As *z* is not an eigenvalue of $A_{0}$, by the above reasoning it is not an eigenvalue of *B*, and hence $z-B$ is injective.

So we conclude that $\sigma (A)= \sigma (B)$ and $\sigma _{\mathrm{ess}}(A_{0})=\sigma _{\mathrm{ess}}(B)$. □

If $DG(0)$ is compact, then we can make inferences on the essential spectrum of *A* from the spectrum of $A_{0}$.

#### Theorem 27

*If* $DG(0)$ *is compact*, *then* $\sigma _{\mathrm{ess}}(A) = \sigma _{\mathrm{ess}}(B)$.

#### Proof

We will prove this by working in the dual space. This is possible as $\sigma _{\mathrm{ess}}(A) = \sigma _{\mathrm{ess}}(A^{*})$, which is a consequence of the properties of Fredholm operators [66, Theorem IV.5.14].

On $X^{*}$, $A^{*}=A_{0}^{*}+L^{*}$ due to Lemma 21. As *ℓ* is bounded, $L= \ell DG(0)$ is compact and so is its adjoint $L^{*}$ due to Schauder’s theorem [66, Theorem III.4.10]. Hence $A^{*}$ is a compact perturbation of $A_{0}^{*}$. One of the defining properties of Weyl’s essential spectrum is that it is invariant under compact perturbations [66, Theorem IV.5.35].

So we conclude that

$$ \sigma _{\mathrm{ess}}(A) = \sigma _{\mathrm{ess}}\bigl(A^{*} \bigr)=\sigma _{\mathrm{ess}}\bigl(A_{0}^{*}\bigr)= \sigma _{\mathrm{ess}}(A_{0})=\sigma _{\mathrm{ess}}(B). $$

 □

In case *B* is the diffusion operator, its essential spectrum is empty, see Lemma 40. This means that also the essential spectrum of *A* is empty when $DG(0)$ is compact.

For computation of the eigenvalues, we follow Engel and Nagel [58]. We introduce the family of operators $K^{z}:Y\rightarrow Y$, $H^{z}:X \rightarrow X$ and $W^{z}: X \rightarrow Y$ parametrized by $z\in \mathbb{C}$, defined as

30 $$ \begin{aligned} &K^{z} y:= DG(0) \bigl(y e^{z\theta }\bigr), \\ &\bigl(H^{z}\varphi \bigr) (\theta ):= \int _{\theta }^{0} e^{z(\theta -s)} \varphi ( \theta )\,ds, \\ &W^{z}\varphi:= \varphi (0)+DG(0)H^{z}\varphi \end{aligned} $$

for $y \in Y$, $\varphi \in X$ and $\theta \in [-h,0]$. Using these, we can define the characteristic operator $\Delta (z)$

31 $$ \Delta (z) = z-B-K^{z}. $$

Now we formulate the main theorem of this section, which allows us to reduce the computation of the eigenvalues and eigenvectors in *X* to a computation on *Y*.

#### Theorem 28

([58, Proposition VI.6.7])

*For every* $z \in \mathbb{C}$, $\varphi \in \mathcal{R}(z-A)$ *if and only if*

$$ \Delta (z)q = W^{z} \varphi $$

*has a solution* $q\in D(B)$. *Moreover*, $z\in \rho (A)$ *if and only if this* *q* *is unique*. *In that case the resolvent is given by*

$$ \bigl(R(z,A)\psi \bigr) (\theta ) = e^{z\theta }\Delta ^{-1}(z)W^{z}\varphi +\bigl(H^{z} \psi \bigr) (\theta ), $$

*where* $\theta \in [-h,0]$ *and* $\psi \in X$. *Finally*, $\psi \in D(A)$ *is an eigenvector corresponding to* $\lambda \in \sigma _{p}(A)$ *if and only if* $\psi (\theta )=e^{\lambda \theta }q$, *where* $q\in D(B)$ *is nontrivial and satisfies*

$$ \Delta (\lambda )q=0. $$

### Hopf bifurcation

We are interested in the nonlinear behaviour of (ADDE). In this section we develop techniques to compute the first Lyapunov coefficient for (Andronov-)Hopf bifurcations. These techniques can be extended to other local bifurcations, but we do not address those here. In this section, we follow the methods from van Gils et al. [33].

Suppose that $\sigma (A)$ contains a pair of simple purely imaginary eigenvalues $\lambda = \pm i\omega $ with $\omega >0$ and no other eigenvalues on the imaginary axis. Let $\psi \in X$ be the corresponding eigenvector of *A* and $\psi ^{\odot } \in X^{\odot }$ be the corresponding eigenvector of $A^{\odot }$, respectively,

32 $$ A\psi = i \omega \psi ,\qquad A^{\odot }\psi ^{\odot }= i \omega \psi ^{\odot }. $$

We normalise these vectors such that

33 $$ \bigl\langle \psi ^{\odot },\psi \bigr\rangle =1. $$

The centre subspace $X_{0}$ is spanned by the basis $\Psi =\{\psi ,\bar{\psi }\}$ of eigenvectors corresponding to the critical eigenvalues of *A*. Here *ψ̄* denotes the complex conjugate of *ψ*.

In order to extend this to the nonlinear setting, we need a (locally) invariant critical centre manifold $W^{c}_{\mathrm{loc}}$, which is tangent to $X_{0}$ at the equilibrium at the origin. From [6], we get a general result on the existence of this centre manifold.

#### Theorem 29

([6, Theorem 41])

*If the strongly continuous semi*-*group* *S* *generated by* *B* *is immediately norm continuous*, $X_{0}$ *is finite*-*dimensional*, $\sigma (A)$ *is the pairwise disjoint union of the sets*

$$\begin{aligned} &\sigma _{-}:= \bigl\{ \lambda \in \sigma (A)| \operatorname{Re}\lambda < 0\bigr\} , \\ &\sigma _{0}:= \bigl\{ \lambda \in \sigma (A)| \operatorname{Re}\lambda =0\bigr\} , \\ &\sigma _{+}:= \bigl\{ \lambda \in \sigma (A)| \operatorname{Re}\lambda >0\bigr\} , \end{aligned}$$

*where* $\sigma _{-}$ *is closed and both* $\sigma _{0}$, $\sigma _{+}$ *are compact*, *and if*

$$ \sup_{\lambda \in \sigma _{-}} \operatorname{Re}\lambda < 0 < \inf _{ \lambda \in \sigma _{+}} \operatorname{Re}\lambda , $$

*then there exist a* $C^{k}$-*smooth mapping* $\mathcal{C}:X_{0} \rightarrow X$ *and an open neighbourhood* *U* *of the origin in* $X_{0}$ *such that* $\mathcal{C}(0)=0$, $D\mathcal{C}(0)= I_{X_{0} \rightarrow X}$, *the identity mapping*, *and* $\mathcal{W}^{c}_{\mathrm{loc}}=\mathcal{C}(U)$ *is locally positively invariant for* (ADDE) *and contains every solution of* (AIE) *that exists on* $\mathbb{R}$ *and remains sufficiently small for all time*.

The conditions on $\sigma (A)$ can be easily satisfied when $\sigma _{0}$ and $\sigma _{+}$ are composed of finitely many eigenvalues of finite multiplicity. In case *B* is the diffusion operator, it is immediately norm continuous by Lemma 39 and the essential spectrum $\sigma _{ess}(A)=\sigma _{ess}(B)=\emptyset $ by Theorem 27 and Lemma 40. Also, when $B=-\alpha I$, $\alpha >0$, we get that the conditions are likewise satisfied.

If $\zeta \in X_{0}$, then we can write $\zeta = z \psi + \bar{z}\bar{\psi }$ for some $z\in \mathbb{C}$. Using this we can recast $\mathcal{C}(U)$ into the formal expansion $\mathcal{H}:\mathbb{C} \rightarrow W^{c}_{\mathrm{loc}}$:

34 $$ \mathcal{H}(z,\bar{z})= z \psi + \bar{z}\bar{\psi } + \sum _{j+k \geq 2} \frac{1}{j! k!} h_{jk}z^{j} \bar{z}^{k}. $$

Due to Theorem 18, (ADDE) and (AIE) formulations are equivalent. By weak^∗^ differentiation of (AIE) and exploiting the finite dimensionality of $\mathcal{W}^{c}_{\mathrm{loc}}$, one can show that a solution $v\in C(\mathbb{R}^{+};X)$, $v(t)=u_{t}$, of (AIE) satisfies the abstract ODE

35 $$ \dot{v}(t) =j^{-1}\bigl(A^{\odot *} j v(t) + \ell R\bigl(v(t)\bigr)\bigr), $$

where the nonlinearity $R: X\rightarrow Y$ is given by

36 $$ R(\varphi ):=G(\varphi )-DG(0) (\varphi )=\frac{1}{2}D^{2}G(0) ( \varphi ,\varphi )+\frac{1}{6}D^{3}G(0) (\varphi , \varphi ,\varphi )+ \mathcal{O}\bigl( \Vert \varphi \Vert ^{4} \bigr). $$

Let $\zeta (t) = z(t) \psi + \bar{z}(t)\bar{\psi }$ be the projection of $v(t)$ onto the centre subspace $X_{0}$. The function $z(t)$ satisfies a complex ODE which is smoothly equivalent to the Poincaré normal form

37 $$ \dot{z}= i \omega z + c_{1} z \vert z \vert ^{2} + \mathcal{O}\bigl( \vert z \vert ^{4}\bigr), $$

where $z,c_{1} \in \mathbb{C}$. In polar coordinates, $z=r e^{i \theta }$, this is orbitally equivalent to

38 $$ \textstyle\begin{cases} \dot{r}= l_{1} r^{3} + \mathcal{O}( \vert r \vert ^{4}), \\ \dot{\theta } = 1 + \mathcal{O}( \vert r \vert ^{2}), \end{cases} $$

where $l_{1}$ is the *first Lyapunov coefficient* determined by the formula

39 $$ l_{1}=\frac{1}{\omega }\operatorname{Re}(c_{1}). $$

It is well known [67] that in generic unfoldings of (38), $l_{1}<0$ implies that the bifurcation is supercritical and that a stable limit cycle exists near one of the branches. On the other hand, $l_{1}>0$ implies that the bifurcation is subcritical and that an unstable limit cycle exists near one of the branches.

The critical centre manifold $\mathcal{W}^{c}_{\mathrm{loc}}$ has expansion (34), and due to the time-invariance of $\mathcal{W}^{c}_{\mathrm{loc}}$, we have

40 $$ v(t)=\mathcal{H}\bigl(z(t),\bar{z}(t)\bigr). $$

If we differentiate both sides with respect to time and use the abstract ODE (35) for the left-hand side, we obtain the *homological equation*

41 $$ A^{\odot *} j \mathcal{H}(z,\bar{z}) + \ell R\bigl( \mathcal{H}(z,\bar{z})\bigr) = j \mathcal{H}_{z}(z,\bar{z})\dot{z} + j \mathcal{H}_{\bar{z}}(z, \bar{z})\dot{\bar{z}}. $$

We can substitute the expansion of nonlinearity (36), the normal form (37) and the expansion of the critical centre manifold (34) into the homological equation (41) to derive the normal form coefficients. If we equate coefficients of the corresponding powers of *z* and *z̄*, we obtain the following equations:

42 $$ \begin{aligned} &-A^{\odot *}j h_{20}= \ell D^{2}G(0) (\psi ,\psi ), \\ &\bigl(2 i \omega - A^{\odot *}\bigr)j h_{11}= \ell D^{2}G(0) (\psi , \bar{\psi }), \\ &\bigl(i \omega - A^{\odot *}\bigr)j h_{21}= \ell D^{3}G(0) (\psi ,\psi , \bar{\psi })+\ell D^{2}G(0) (h_{20},\bar{\psi }) \\ &\hphantom{\bigl(i \omega - A^{\odot *}\bigr)j h_{21}=}{}+ 2\ell D^{2}G(0) (\psi ,h_{11})-2c_{1}j \psi . \end{aligned} $$

They all have the form

43 $$ \bigl(z - A^{\odot *}\bigr)\varphi ^{\odot *}=\psi ^{\odot *}. $$

Here $z \in \mathbb{C}$ and $\psi ^{\odot *}\in X^{\odot *}$ are given. When $z \in \rho (A)$, then (43) has a unique solution. However, if $z \in \sigma (A)$, then a solution $\varphi ^{\odot *}$ does not necessarily exist for all $\psi ^{\odot *}$. The following lemma, which is equivalent to [33, Lemma 33], provides a condition for solvability.

#### Lemma 30

(Fredholm solvability)

*Let* $z \notin \sigma _{\mathrm{ess}}(A)$. *Then* $z - A^{\odot }:D(A^{\odot })\rightarrow X^{\odot }$ *has a closed range*. *In particular* $(z -A^{\odot *})\varphi ^{\odot *}=\psi ^{\odot *}$ *is solvable for* $\varphi ^{\odot *} \in D(A^{\odot *})$ *given* $\psi \in X^{\odot *}$ *if and only if* $\langle \psi ^{\odot *},\psi ^{\odot }\rangle =0$ *for all* $\psi ^{\odot }\in \mathcal{N}(z-A^{\odot })$.

#### Proof

From the definition of the essential spectrum, $\mathcal{R}(z -A)$ is closed [66, Section IV.5.1], and $\mathcal{R}(z-A^{*})$ is also closed by Banach’s closed range theorem [66, Theorem IV.5.13]. Let $(\psi _{n}^{\odot })_{n\in \mathbb{N}}$ be a sequence in $\mathcal{R}(z-A^{\odot })$ such that $\psi _{n}^{\odot }\rightarrow \psi ^{\odot }\in X^{\odot }$. Then there is a sequence $(\varphi _{n}^{\odot })_{n\in \mathbb{N}}$ in $D(A^{\odot })$ such that

$$ \psi _{n}^{\odot }= \bigl(z-A^{\odot }\bigr)\varphi _{n}^{\odot }= \bigl(z-A^{*}\bigr)\varphi _{n}^{\odot }\quad \forall n \in \mathbb{N}. $$

Hence $\psi _{n}^{\odot }\in \mathcal{R}(z-A^{*})$ for all $n\in \mathbb{N}$, so there exists $\varphi ^{\odot }\in D(A^{*})$ such that $(z-A^{*})\varphi ^{\odot }= \psi ^{\odot }$ and

$$ A^{*}\varphi ^{\odot }= z \varphi ^{\odot }- \bigl(z-A^{*}\bigr)\varphi ^{\odot }= z \varphi ^{\odot }- \psi ^{\odot }\in X^{\odot }. $$

Hence $\varphi ^{\odot }\in D(A^{\odot })$, $(z-A^{\odot })\varphi ^{\odot }= \psi ^{\odot }$ and $\psi ^{\odot }\in \mathcal{R}(z-A^{\odot })$.

Due to Banach’s closed range theorem, $\varphi ^{\odot *}$ is a solution of

$$ \bigl(z-A^{\odot *}\bigr)\varphi ^{\odot *}=\psi ^{\odot *} $$

given $\psi ^{\odot *}$ if and only if

$$ \bigl\langle \psi ^{\odot *}, \psi ^{\odot }\bigr\rangle =0\quad \forall \psi ^{\odot }\in \mathcal{N}\bigl(z-A^{\odot }\bigr). $$

 □

We now return to equations (42). As $\{0,2i \omega \} \subset \rho (A)=\rho (A^{\odot })$, we can use the resolvent of $A^{\odot *}$ to solve the first two equations. However, $i \omega \in \sigma (A)$, so for the last equation of (42) we need to use the theorem above. The corresponding eigenspace $\mathcal{N}(A^{*}-\lambda )$ is spanned by $\psi ^{\odot }$, so we can compute for the normal form coefficient by

44 $$ \begin{aligned} &j h_{20}= R\bigl(0,A^{\odot *} \bigr)\ell D^{2}G(0) (\psi ,\psi ), \\ &j h_{11}= R\bigl(2i \omega ,A^{\odot *}\bigr)\ell D^{2}G(0) (\psi ,\bar{\psi }), \\ &c_{1}=\frac{1}{2}\bigl\langle \ell D^{3}G(0) (\psi ,\psi ,\bar{\psi })+ \ell D^{2}G(0) (h_{20},\bar{ \psi })+ 2\ell D^{2}G(0) (\psi ,h_{11}), \psi ^{\odot }\bigr\rangle . \end{aligned} $$

We are not yet able to compute the normal form coefficient explicitly as we do not have an explicit representation of $\psi ^{\odot }$ or a representation of the resolvent of $A^{\odot *}$. However, we resolve this by using spectral projections.

Let $P^{\odot }$ and $P^{\odot *}$ be the spectral projections on $X^{\odot }$ and $X^{\odot *}$ corresponding to some eigenvalue *λ*, respectively. Then $P^{\odot *}\varphi ^{\odot *} = \nu j \psi $ for some $\nu \in \mathbb{C}$ and

$$ \bigl\langle \varphi ^{\odot *}, \psi ^{\odot }\bigr\rangle =\bigl\langle \varphi ^{ \odot *}, P^{\odot }\psi ^{\odot }\bigr\rangle =\bigl\langle P^{\odot *}\varphi ^{ \odot *}, \psi ^{\odot }\bigr\rangle =\nu \bigl\langle j\psi , \psi ^{\odot } \bigr\rangle =\nu . $$

Hence we seek to determine *ν*. From the Dunford integral representation it follows that

45 $$ P^{\odot *}\varphi ^{\odot *} = \frac{1}{2\pi i} \oint _{\partial C_{\lambda }} R\bigl(z,A^{\odot *}\bigr)\varphi ^{\odot *}\,dz= \nu j \psi , $$

where $C_{\lambda }$ is a sufficiently small open disk centred at *λ* and $\partial C_{\lambda }$ is its boundary. The element on the left in the pairing (44) is of the form $\varphi ^{\odot *} = \ell y$, $y\in Y$. In this case we can reduce $R(z,A^{\odot *})\varphi ^{\odot *}$ to $\Delta ^{-1}(z)y$ by virtue of the following theorem.

#### Theorem 31

*Suppose that* $z \in \rho (A)$. *For each* $y\in Y$, *the function* $\varphi \in X$, *defined as* $\varphi (\theta ) : = e^{z \theta } \Delta ^{-1}(z) y$ *for* $\theta \in [-h,0]$, *is the unique solution in* $\{\varphi \in C^{1}([-h,0];Y)| \varphi (0)\in D(B)\}$ *of the system*

46 $$ \textstyle\begin{cases} (z-B)\varphi (0) - DG(0)\varphi = y, \\ z \varphi -\dot{\varphi } = 0. \end{cases} $$

*Moreover*, $\varphi ^{\odot *} = j\varphi $ *is the unique solution in* $D(A^{\odot *})$ *of* $(z-A^{\odot *})\varphi ^{\odot *} = \ell y $.

#### Proof

Since $z \in \rho (A)$, by Theorem 28 it follows that $\Delta ^{-1}(z)$ exists. We start by showing that *φ* as defined above solves (46). Clearly, $\varphi \in C^{1}([-h,0];Y)$ and $\varphi (0)=\Delta ^{-1}(z)y\in D(B)$. Recall from the definition of $K^{z}$ that for $q\in Y$, $K^{z} q = DG(0)q e^{z \theta }$. Therefore,

$$ (z-B)\varphi (0) - DG(0)\varphi = (z-B)\Delta ^{-1}(z)y - K^{z} \Delta ^{-1}(z) y = y. $$

Finally, by differentiating *φ*, we see that it satisfies the second equation in (46).

When $\varphi (0)\in D(B)$, then $j_{Y} \varphi (0) \in D(B^{\odot *})$, because for all $y^{\odot }\in D(B^{\odot })$

$$ \bigl\langle j_{Y} B\varphi (0), y^{\odot }\bigr\rangle = \bigl\langle y^{\odot }, B \varphi (0) \bigr\rangle = \bigl\langle B^{\odot }y^{\odot }, \varphi (0) \bigr\rangle = \bigl\langle j_{Y} \varphi (0), B^{\odot }y^{\odot }\bigr\rangle . $$

Then Corollary 23 implies that $j \varphi \in D(A^{\odot *})$.

$$ \bigl(z-A^{\odot *}\bigr)\varphi ^{\odot *} = \bigl(j_{Y}(z-B) \varphi (0) - j_{Y} DG(0) \varphi ,z \varphi -\dot{\varphi } \bigr)=(j_{Y} y,0)= \ell y. $$

However, by Theorem 28, $\rho (A^{\odot *}) = \rho (A)$, so $\varphi ^{\odot *} = j \varphi $ is the unique solution of $(z-A^{\odot *})\varphi ^{\odot *} = \ell y$. Consequently, *φ* itself is the unique solution in $\{\varphi \in C^{1}([-h,0];Y)| \varphi (0)\in D(B)\}$. □

Now given that we can compute the resolvent $\Delta ^{-1}(z)$ and the Fréchet derivatives of *G*, we have a method to compute the centre manifold coefficients $h_{20}$ and $h_{11}$, and the first Lyapunov coefficient $l_{1} = \frac{1}{\omega }\operatorname{Re}c_{1}$:

47 $$ \begin{aligned} &h_{20}(\theta )= \Delta ^{-1}(0) D^{2}G(0) (\psi ,\psi ), \\ &h_{11}(\theta )= e^{2i \omega \theta }\Delta ^{-1}(2 i \omega ) D^{2}G(0) ( \psi ,\bar{\psi }), \\ &c_{1} \psi (\theta )= \frac{1}{4\pi i} \oint _{\partial C_{\lambda }} e^{z\theta }\Delta ^{-1}(z) \bigl(D^{3}G(0) (\psi ,\psi , \bar{\psi }) + D^{2}G(0) (h_{20},\bar{\psi }) \\ &\hphantom{c_{1} \psi (\theta )=}{} + 2 D^{2}G(0) (\psi ,h_{11})\bigr)\,dz. \end{aligned} $$

## Characterisation of the spectrum

In this section we return to the neural field as derived in Sect. 1.3. For certain choices we can derive some explicit conditions for the spectrum and find an explicit expression for the resolvent.

We take $Y=C(\Omega )$ with $\Omega = [-1,1]$ and use the (ADDE) formulation of Sect. 2

ADDE $$ \textstyle\begin{cases} \dot{u}(t)=Bu(t)+G(u_{t}), \\ u_{0}=\varphi \in X, \end{cases} $$

where $B: D(B)\rightarrow Y$ and $G:X\rightarrow Y$ are defined as

$$\begin{aligned} &B q:= d q'' - \alpha q, \\ &D(B):= \bigl\{ q\in Y | q\in C^{2}(\Omega ), q'( \partial \Omega )=0 \bigr\} , \\ &G(\varphi ):= \alpha \int _{\Omega } J\bigl(x,x'\bigr)S\bigl(\varphi \bigl(t-\tau \bigl(x,x'\bigr),x'\bigr)\bigr)\,dx'. \end{aligned}$$

Here, we assume that $d \geq 0$, $\alpha >0$, *J* and *τ* are continuous functions and $S \in C^{\infty }(\mathbb{R})$, with $S(0)=0$ and $S'(0)\neq 0$. The assumption $S(0)=0$ makes sure we have an equilibrium at $u\equiv 0$. We interpret *u* as the deviation from this physiological resting state. This interpretation then makes for cleaner notation.

We have the following properties for *G* and its derivatives.

### Lemma 32

([33, Lemma 3, Proposition 11])

*G* *is compact*, *globally Lipschitz continuous and* *k* *times Fréchet differentiable for any* $k\in \mathbb{N}$. *Furthermore*, *the* *kth Fréchet derivative of* *G* *at* $\psi \in X$, $D^{k}G(\psi ): X^{k} \rightarrow Y$, *is compact and given by*

$$\begin{aligned} &\bigl(D^{k}G(\psi ) (\varphi _{1},\ldots , \varphi _{k})\bigr) (x)\\ &\quad = \alpha \int _{ \Omega } \Biggl[ J\bigl(x,x' \bigr)S^{(k)}\bigl(\psi \bigl(-\tau \bigl(x,x' \bigr),x'\bigr)\bigr) \prod_{m=1}^{k}\bigl(\varphi _{m}\bigl(-\tau \bigl(x,x'\bigr),x' \bigr)\bigr) \Biggr] \,dx'. \end{aligned}$$

As $DG(0)$ is compact, we can find, due to Theorem 27 and Lemma 40, that the essential spectrum of the linearisation *A* is given by

48 $$ \sigma _{\mathrm{ess}}(A) = \textstyle\begin{cases} \emptyset & d>0, \\ \{-\alpha \} & d=0. \end{cases} $$

We want to be able to compute the eigenvalues, eigenvectors and resolvent for specific choices of *J* and *τ*. We take *J* as a sum of exponentials and *τ* as a constant delay plus a finite propagation speed, which we can normalise to 1 by scaling time.

$$\begin{aligned} &J\bigl(x,x'\bigr):= \sum_{j=1}^{N} \eta _{j} e^{-\mu _{j} \vert x-x' \vert }, \\ &\tau \bigl(x,x'\bigr):= \tau ^{0} + \bigl\vert x-x' \bigr\vert , \end{aligned}$$

where we take $\tau ^{0} \geq 0$ and $\eta _{j} \neq 0$ for $j \in \{1,\ldots ,N\}$.

Due to Theorem 28, we have that *λ* is an eigenvalue and *ψ* an eigenvector if and only if $\psi (\theta ) = q e^{\lambda \theta }$ and $q \in D(B)$ satisfies the characteristic equation (CE).

CE $$ \Delta (\lambda )q=\bigl(\lambda - B - K^{\lambda }\bigr)q=0, $$

where in this case $K^{z}: Y \rightarrow Y$ is a parametrized family of operators for $z\in \mathbb{C}$ defined as follows:

49 $$ \begin{aligned} &K^{z}:= \sum _{j=1}^{N} K^{z}_{j}, \\ &K_{j}^{z} y(x):= c_{j}(z) \int _{-1}^{1} e^{-k_{j}(z) |x-x'|} y \bigl(x'\bigr)\,dx', \end{aligned} $$

where $c_{j}(z) := S'(0) \alpha \eta _{j} e^{-\tau ^{0} z}\neq 0$ and $k_{j}(z) := \mu _{j} + z$.

The case without diffusion, i.e. $d=0$, has already been extensively studied [33, 34], so in this section we develop formulas for the eigenvalues, eigenvectors and resolvent with nontrivial diffusion, i.e. $d>0$.

For the following section, we adopt the notational convention that bold-faced variables correspond to vectors $\mathbf{a}=(a_{1} \cdots a_{n})^{T}$ where its length is clear from the context.

### Eigenvalues

So we are looking for nontrivial solutions $q \in D(B)$ of

CE $$ \bigl(z-B- K^{z}\bigr)q=0. $$

As this is a mixed differential-integral equation, it is in general hard to solve. We will use the method of Dijkstra et al. [34] to convert (CE) into a differential equation (ODE), which we can solve. Then substituting the general solution of (ODE) back into (CE) yields appropriate conditions on *q*. This is possible due to the following observations.

#### Lemma 33

*All solutions of* (CE) *are* $C^{\infty }(\Omega )$.

#### Proof

As $q\in C^{2}(\Omega )$ and the range of $K^{z}$ is contained in $C^{3}(\Omega )$, we have that $B q \in C^{2}(\Omega )$, which means that $q\in C^{4}(\Omega )$. By induction, we conclude that $q \in C^{\infty }(\Omega )$. □

Differentiating the kernel functions in the (CE) in the distributional sense yields, for $j\in \{1,\ldots , N\}$,

$$ \frac{\partial ^{2}}{\partial x^{2}}e^{-k_{j}(z)|x-x'|}= \bigl[ k_{j}^{2}(z)-2k_{j}(z) \delta \bigl(x-x'\bigr) \bigr]e^{-k_{j}(z)|x-x'|}. $$

So we define the differential operator $L_{j}^{z}$ for $j \in \{1,\ldots , N\}$:

$$ L_{j}^{z}:= k_{j}^{2}(z)- \partial _{x}^{2}. $$

For this operator $L_{j}$, we have that for $j\in \{1,\ldots , N\}$

$$ L_{j}^{z} K_{j}^{z} q = 2 c_{j}(z) k_{j}(z) q. $$

Hence, by applying the operator $L^{z} = \prod_{p=1}^{N} L^{z}_{p}$ to (CE), we end up with an ordinary differential equation (ODE)

ODE $$ L^{z}\Delta (z)q=(z-B)\prod _{p=1}^{N} L_{p}^{z} q - 2 \sum_{j=1}^{N} c_{j}(z) k_{j}(z) \prod_{\substack{p=1\\ p\neq j}}^{N} L_{p}^{z} q =0. $$

This differential equation has a characteristic polynomial corresponding to exponential solutions $e^{\rho x}$

50 $$ P^{z}(\rho ):=\bigl(\alpha +z-d\rho ^{2}\bigr)\prod_{p=1}^{N} \bigl(k_{p}(z)^{2}- \rho ^{2}\bigr)-2 \sum _{j=1}^{N} c_{j}(z)k_{j}(z) \prod_{ \substack{p=1\\ p\neq j}}^{N} \bigl(k_{p}(z)^{2}- \rho ^{2}\bigr). $$

$P^{z}$ is an even polynomial of order $2(N+1)$. Assuming that *z* is such that $P^{z}$ has exactly $2(N+1)$ distinct roots $\pm \rho _{1}(z),\ldots ,\pm \rho _{N+1}(z)$, the general solution *q* of (ODE) is a linear combination of exponentials $e^{\pm \rho _{j} x}$:

51 $$ q(x):=\sum_{m=1}^{N+1} \bigl[a_{m} \cosh \bigl(\rho _{m}(z) x \bigr)+b_{m} \sinh \bigl(\rho _{m}(z) x\bigr) \bigr]). $$

Writing *q* as a linear combination of cosine hyperbolic and sine hyperbolic leads to cleaner notation below.

Before we substitute (51) back into (CE), we first prove two lemmas.

#### Lemma 34

*If the characteristic polynomial* $P^{z}(\rho )$ *has* $2(N+1)$ *distinct roots*, *then* $\rho _{m}(z)\neq 0$ *for all* $m\in \{1,\ldots , N+1\}$ *and* $k_{j}(z)\neq 0$ *for all* $j \in \{1,\ldots , N\}$.

#### Proof

If $P^{z}(\rho )$ has $2(N+1)$ distinct roots $\pm \rho _{1}(z), \ldots , \pm \rho _{N+1}(z)$, then $\rho _{m}(z)$ is distinct from $- \rho _{m}(z)$ and hence $\rho _{m}(z) \neq 0$ for $m\in \{1,\ldots , N+1\}$.

Let without loss of generality $k_{1}(z)=0$. In that case the characteristic polynomial becomes

$$ P^{z}(\rho )=\rho ^{2}\bigl(\alpha +z-d\rho ^{2}\bigr) \prod_{p=2}^{N} \bigl(k_{p}(z)^{2}- \rho ^{2}\bigr)-2 \rho ^{2} \sum_{j=2}^{N} c_{j}(z)k_{j}(z) \prod_{ \substack{p=2\\ p\neq j}}^{N} \bigl(k_{p}(z)^{2}-\rho ^{2}\bigr). $$

So $\rho =0$ is a root of $P^{z}$. Hence we conclude by contradiction that $k_{j}(z)\neq 0$ for all $j \in \{1,\ldots , N\}$. □

Define the set $\mathcal{L}$ as follows:

52 $$ \mathcal{L}:=\bigl\{ z\in \mathbb{C}| \exists j \in \{1,\ldots N \}, m\in \{1, \ldots , N+1\} \text{ such that } k_{j}(z)= \pm \rho _{m}(z) \bigr\} . $$

#### Lemma 35

*If characteristic polynomial* $P^{z}$ *has* $2(N+1)$ *distinct roots*, *then*

$$ \mathcal{L}=\bigl\{ z\in \mathbb{C}| \exists j,p \in \{1,\ldots N\}, j\neq p \textit{ such that } k_{j}^{2}(z)= k_{p}^{2}(z) \bigr\} . $$

#### Proof

We have that $z\in \mathcal{L}$ if and only if $P^{z}(k_{j}(z)) = 0$ for some $j\in \{1,\ldots , N\}$.

$$ P^{z}\bigl(k_{j}(z)\bigr)=-2c_{j}(z)k_{j}(z) \prod_{\substack{p=1\\ p\neq j}}^{N} \bigl(k_{p}^{2}(z)-k_{j}^{2}(z) \bigr). $$

Hence $P^{z}(k_{j}(z)) = 0$ if and only if $k_{j}^{2}(z)= k_{p}^{2}(z)$ for some $p \in \{1,\ldots N\}$, $j\neq p$. □

For $z\notin \mathcal{L}$, we can rewrite $P^{z}(\rho _{m})$ as follows:

$$ P^{z}(\rho _{m}) = \Biggl[\alpha + z - d \rho _{m}^{2} - \sum_{j=1}^{N} \frac{2c_{j}(z)k_{j}(z)}{k_{j}^{2}(z)-\rho _{m}^{2}(z)} \Biggr]\prod_{p=1}^{N} \bigl(k_{p}^{2}(z)- \rho _{m}^{2}(z) \bigr)=0. $$

We can divide out the product to conclude that, for $m\in \{1,\ldots ,N+1\}$ and $j\in \{1,\ldots , N\}$,

53 $$ \alpha + z - d \rho _{m}^{2} - \sum _{j=1}^{N} \frac{2c_{j}(z)k_{j}(z)}{k_{j}^{2}(z)-\rho _{m}^{2}(z)}=0. $$

Next we find formulas for $K_{j}^{z}\cosh (\rho _{m}(z)x)$ and $K_{j}^{z}\sinh (\rho _{m}(z)x)$. To compute these integrals, we split the interval $[-1,1]$ into the intervals $[-1,x]$ and $[x,1]$. On these intervals $e^{-k|x-x'|}$ is a $C^{1}$ function in $x'$, so we can compute the following anti-derivatives for these smooth branches:

54 $$ \begin{aligned} &\int ^{x'} e^{-k|x-s|} \cosh (\rho s)\,ds \\ &\quad = \textstyle\begin{cases} e^{-k|x-x'|} \frac{( k \cosh (\rho x') - \rho \sinh (\rho x'))}{k^{2}-\rho ^{2}} + \text{const}. & -1\leq x' < x \leq 1, \\ e^{-k|x-x'|} \frac{(-k \cosh (\rho x') - \rho \sinh (\rho x'))}{k^{2}-\rho ^{2}} + \text{const}. & -1\leq x < x' \leq 1, \end{cases}\displaystyle \\ &\int ^{x'} e^{-k|x-s|} \sinh (\rho s)\,ds \\ &\quad = \textstyle\begin{cases} e^{-k|x-x'|} \frac{( k \sinh (\rho x') - \rho \cosh (\rho x'))}{k^{2}-\rho ^{2}} + \text{const}. & -1\leq x' < x \leq 1, \\ e^{-k|x-x'|} \frac{(-k \sinh (\rho x') - \rho \cosh (\rho x'))}{k^{2}-\rho ^{2}} + \text{const}. & -1\leq x < x' \leq 1. \end{cases}\displaystyle \end{aligned} $$

Using these anti-derivatives, we can evaluate the integrals $K_{j}^{z}\cosh (\rho _{m}(z)x)$ and $K_{j}^{z}\sinh (\rho _{m}(z)x)$. For clarity, we omit the dependence on *z* in the remainder of this section.

$$\begin{aligned} &K_{j}\cosh (\rho _{m} x)= \frac{2 c_{j} k_{j}\cosh (\rho _{m} x)-2 c_{j} e^{-k_{j}}\cosh (k_{j} x)(k_{j}\cosh (\rho _{m})+\rho _{m}\sinh (\rho _{m}))}{k_{j}^{2}-\rho _{m}^{2}}, \\ &K_{j}\sinh (\rho _{m} x)= \frac{2 c_{j} k_{j}\sinh (\rho _{m} x)-2 c_{j} e^{-k_{j}}\sinh (k_{j} x)(\rho _{m}\cosh (\rho _{m})+k_{j}\sinh (\rho _{m}))}{k_{j}^{2}-\rho _{m}^{2}}. \end{aligned}$$

Now we are ready to substitute the general solution *q* of (ODE), (51), back into (CE):

55 $$ \begin{aligned} &\sum_{m=1}^{N} \bigl[a_{m}\cosh (\rho _{m} x)+b_{m} \sinh (\rho _{m} x) \bigr] \Biggl[\bigl(\alpha +z+d\rho _{m}^{2}\bigr)+\sum_{j=1}^{N} \frac{2 c_{j} k_{j}}{k_{j}^{2}-\rho _{m}^{2}} \Biggr] \\ &\quad {}+\sum_{j=1}^{N} c_{j} e^{-k_{j}} \Biggl[-\cosh (k_{j} x)\sum _{m=1}^{N+1} a_{m} \frac{k_{j}\cosh (\rho _{m})+\rho _{m}\sinh (\rho _{m})}{k_{j}^{2}-\rho _{m}^{2}} \\ &\quad {} -\sinh (k_{j} x)\sum_{m=1}^{N+1} b_{m} \frac{\rho _{m}\cosh (\rho _{m})+k_{j}\sinh (\rho _{m})}{k_{j}^{2}-\rho _{m}^{2}} \Biggr]=0. \end{aligned} $$

Due to the characteristic equation (53), the first line in equation (55) vanishes. When $z\notin \mathcal{L}$, $\cosh (k_{j} x)$ and $\sinh (k_{j} x)$ for $j \in \{1,\ldots , N\}$ are linearly independent. Hence the second line vanishes if and only if $S^{z,\mathrm{even}}\mathbf{a}=S^{z,\mathrm{odd}}\mathbf{b}=\mathbf{0}$, where matrices $S^{z,\mathrm{even}}$ and $S^{z,\mathrm{odd}}$ are defined as follows:

56 $$ \begin{aligned} &S^{z,\mathrm{even}}_{j,m}:= \frac{k_{j}\cosh (\rho _{m})+\rho _{m}\sinh (\rho _{m})}{k_{j}^{2}-\rho _{m}^{2}}, \\ &S^{z,\mathrm{odd}}_{j,m}:= \frac{\rho _{m}\cosh (\rho _{m})+k_{j}\sinh (\rho _{m})}{k_{j}^{2}-\rho _{m}^{2}} \end{aligned} $$

for $j\in \{1,\ldots , N\}$ and $m \in \{1, \ldots , N+1\}$.

As $q\in D(B)$, we also need to take the boundary conditions into account as

57 $$ q'(\pm 1) = \sum_{m=1}^{N+1} \bigl[b_{m} \rho _{m} \cosh (\rho _{m}) \pm a_{m} \rho _{m} \sinh (\rho _{m}) \bigr]=0. $$

To satisfy the boundary conditions, we augment the matrices $S^{z,\mathrm{even}}$ and $S^{z,\mathrm{odd}}$ as follows:

58 $$ \begin{aligned} &S^{z,\mathrm{even}}_{N+1,m}:=\rho _{m} \sinh (\rho _{m}), \\ &S^{z,\mathrm{odd}}_{N+1,m}:=\rho _{m} \cosh (\rho _{m}). \end{aligned} $$

Now we have square matrices $S^{z,\mathrm{even}}, S^{z,\mathrm{odd}} \in \mathbb{C}^{(N+1)\times (N+1)}$. There exists a nontrivial solution $q\in D(B)$ of (CE) if and only if $\det (S^{z,\mathrm{even}})=0$ or $\det (S^{z,\mathrm{odd}})=0$.

#### Theorem 36

*Suppose that* $\det (P^{\lambda }(\rho ))$ *has* $2(N+1)$ *distinct roots and* $\lambda \notin \mathcal{L}$ *for some* $\lambda \in \mathbb{C}$, *then we have that* $\lambda \in \sigma _{p}(A)$ *if and only if* $\det (S^{\lambda ,\mathrm{even}})\det (S^{\lambda ,\mathrm{odd}})=0$.

*When* $\det (S^{\lambda ,\mathrm{even}})=0$, *the corresponding eigenvector* $\psi \in X$ *is given by*

59 $$ \psi (\theta ) (x):=e^{\lambda \theta }\sum_{m=1}^{N+1}a_{m} \cosh \bigl( \rho _{m}(\lambda ) x\bigr), $$

*where* **a** *is a vector in the nullspace of* $S^{\lambda ,\mathrm{even}}$.

*When* $\det (S^{\lambda ,\mathrm{odd}})=0$, *the corresponding eigenvector* $\psi \in X$ *is given by*

60 $$ \psi (\theta ) (x):=e^{\lambda \theta }\sum_{m=1}^{N(N+1)}b_{m} \sinh \bigl( \rho _{m}(\lambda ) x\bigr), $$

*where* **b** *is a vector in the nullspace of* $S^{\lambda ,\mathrm{odd}}$.

#### Proof

Let $q\in D(B)$ be a solution of (CE) for some $\lambda \in \mathbb{C}$. Then, by Theorem 33, $q\in C^{\infty }$, so it is also a solution of (ODE).

Conversely, let *q* be a solution of (ODE). As $\det (P^{\lambda }(\rho ))$ has $2(N+1)$ distinct roots, *q* is of the form (51). Due to (55) and (57), it is a solution of (CE) if and only if $\det (S^{\lambda ,\mathrm{even}})\det (S^{\lambda ,\mathrm{odd}})=0$. □

We will call an eigenvalue ‘even’, respectively ‘odd’, when $\det (S^{\lambda ,\mathrm{even}})=0$, respectively $\det (S^{\lambda ,\mathrm{odd}})=0$.

### Resolvent

Due to Theorem 31, to compute the normal form coefficients, we need a representation of $\Delta ^{-1}(z)y$. It is defined for $z\in \rho (A)$ as the unique solution $q \in D(B)$ of the resolvent equation (RE)

RE $$ \Delta (z)q= \bigl(z-B-K^{z}\bigr)q =y. $$

We can find an explicit form for this resolvent using a variation-of-constants ansatz when $z\notin \mathcal{S}$, which is defined as follows:

61 $$ \mathcal{S}:=\sigma (B)\cup \mathcal{L}\cup \bigl\{ z \in \mathbb{C}|P^{z}( \rho )\text{ has less than } 2(N+1)\text{ distinct zeros} \bigr\} $$

with $\mathcal{L}$ as in (52).

#### Theorem 37

*For* $z\in \rho (A)$ *with* $z\notin \mathcal{S}$, *the unique solution* $q\in D(B)$ *of* (RE) *is given by*

62 $$ q(x):=R(z,B)y(x)+\sum_{m=1}^{N+1} \bigl[a_{m}(x) \cosh \bigl(\rho _{m}(z) x \bigr)+b_{m}(x) \sinh \bigl(\rho _{m}(z) x\bigr) \bigr], $$

*where* $R(z,B)$ *is the resolvent operator of* *B* *as in* (97) *and* $\mathbf{a}(x)$ *and* $\mathbf{b}(x)$ *as in* (79)

#### Proof

Our variation-of-constants ansatz *q* needs to satisfy three conditions. It must solve (RE), $\Delta (z)q=y$, it must satisfy the boundary conditions $(q)'(\pm 1)=0$ and the regularity condition $q \in C^{2}(\Omega )$. When we found some $a_{m}(x)$, $b_{m}(x)$ such that *q* satisfies these conditions, we have found the resolvent as it is unique due to Theorem 28. As $R(z,B)$ maps into $D(B)$, the regularity condition is satisfied when $\mathbf{a}(x), \mathbf{b}(x) \in C^{2}(\Omega )$. For this proof, we suppress the dependencies on *z*.

To aid in the calculation of $\Delta (z)q$, we first compute some integrals up front. We can integrate by parts by splitting the interval $[-1,1]$ into $[-1,x)$ and $(x,1]$ and using the anti-derivatives in (54) to end up with

63 $$\begin{aligned} &K_{j} a_{m}(x)\cosh(\rho _{m} x) \\ &\quad = a_{m}(x) \cosh (\rho _{m} x)\frac{2 c_{j} k_{j} }{k_{j} ^{2}-\rho _{m}^{2}} \\ &\qquad {}+c_{j} e^{-k_{j} (1+x)}a_{m}(-1)S^{z,\mathrm{even}}_{j,m}+c_{j} e^{-k_{j} (1-x)}a_{m}(1)S^{z,\mathrm{even}}_{j,m} \\ \begin{aligned}&\qquad {}-c_{j} \int _{-1}^{1} \frac{a_{m}'(x')}{k_{j} ^{2}-\rho _{m}^{2}}e^{-k_{j} |x-x'|} \bigl(\mathrm{sgn}\bigl(x-x'\bigr)k_{j} \cosh \bigl( \rho _{m} x'\bigr) - \rho _{m} \sinh \bigl(\rho _{m} x'\bigr) \bigr)\,dx', \\ &K_{j} b_{m}(x)\sinh(\rho _{m} x)\end{aligned} \\ &\quad = b_{m}(x)\sinh (\rho _{m} x) \frac{2 c_{j} k_{j} }{k_{j} ^{2}-\rho _{m}^{2}} \\ &\qquad {}-c_{j} e^{-k_{j} (1+x)}b_{m}(-1)S^{z,\mathrm{odd}}_{j,m}+c_{j} e^{-k_{j} (1-x)}b_{m}(1)S^{z,\mathrm{odd}}_{j,m} \\ &\qquad {}-c_{j} \int _{-1}^{1} \frac{b_{m}'(x')}{k_{j} ^{2}-\rho _{m}^{2}}e^{-k_{j} |x-x'|} \bigl(\mathrm{sgn}\bigl(x-x'\bigr)k_{j} \sinh \bigl( \rho _{m} x'\bigr) - \rho _{m} \cosh \bigl(\rho _{m} x'\bigr) \bigr)\,dx'. \end{aligned}$$

Now we substitute ansatz (62) into (RE) and collect the terms. Using the above calculations and the fact that $(z-B)R(z,B)y=y$, we have that

64a $$\begin{aligned} 0={}&\sum_{m=1}^{N+1} \bigl[a_{m}(x)\cosh (\rho _{m} x)+b_{m}(x) \sinh ( \rho _{m} x) \bigr] \\ &{}\times \Biggl[\bigl(\alpha +z-d \rho _{m}^{2}(z)\bigr)-\sum_{j=1}^{N} \frac{2 c_{j} k_{j} }{k_{j} ^{2}-\rho _{m}^{2}} \Biggr] \end{aligned}$$

64b $$\begin{aligned} &{}-\sum_{m=1}^{N+1}\,d\bigl[ \bigl(a_{m}''(x)+2\rho _{m} b_{m}'(x)\bigr)\cosh ( \rho _{m} x)+\bigl(b_{m}''(x)+2 \rho _{m} a_{m}'(x)\bigr)\sinh (\rho _{m} x) \bigr] \end{aligned}$$

64c $$\begin{aligned} &{}-\sum_{j=1}^{N} c_{j} e^{-k_{j} (1+x)} \Biggl[\sum_{m=1}^{N+1}a_{m}(-1)S^{z,\mathrm{even}}_{j,m}- \sum_{m=1}^{N+1}b_{m}(-1)S^{z,\mathrm{odd}}_{j,m} \Biggr] \end{aligned}$$

64d $$\begin{aligned} &{}-\sum_{j=1}^{N} c_{j} e^{-k_{j} (1-x)} \Biggl[\sum_{m=1}^{N+1}a_{m}(1)S^{z,\mathrm{even}}_{j,m}+ \sum_{m=1}^{N+1}b_{m}(1)S^{z,\mathrm{odd}}_{j,m} \Biggr] \end{aligned}$$

64e $$\begin{aligned} &{}-\sum_{j=1}^{N}c_{j} \int _{-1}^{1}e^{-k_{j} |x-x'|} \Biggl[R(z,B)y \bigl(x'\bigr) \\ &{} -\sum_{m=1}^{N+1} \frac{a_{m}'(x')}{k_{j} ^{2}-\rho _{m}^{2}} \bigl(\mathrm{sgn}\bigl(x-x' \bigr)k_{j} \cosh \bigl(\rho _{m} x' \bigr) - \rho _{m} \sinh \bigl(\rho _{m} x'\bigr) \bigr) \\ &{} -\sum_{m=1}^{N+1} \frac{b_{m}'(x')}{k_{j} ^{2}-\rho _{m}^{2}} \bigl(\mathrm{sgn}\bigl(x-x' \bigr)k_{j} \sinh \bigl(\rho _{m} x' \bigr) - \rho _{m} \cosh \bigl(\rho _{m} x'\bigr) \bigr) \Biggr]\,dx'. \end{aligned}$$

We have that the above equation vanishes when all the terms within square brackets vanish. Term (64a) vanishes naturally due to characteristic equation in (53) as $z\notin \mathcal{L}$.

As $R(z,B)$ maps into $D(B)$, the boundary condition $q'(\pm 1)=0$ reduces to

65 $$ \sum_{m=1}^{N+1} \bigl[\bigl(a_{m}'(\pm 1)+\rho _{m} b_{m}(\pm 1)\bigr) \cosh ( \rho _{m})\pm \bigl(b_{m}'(\pm 1)+\rho _{m} a_{m}(\pm 1)\bigr) \sinh (\rho _{m}) \bigr]=0 .$$

We can split equation (65) into three sufficient equations:

66a $$\begin{aligned} &\sum_{m=1}^{N+1} \bigl[a_{m}'( \pm 1)\cosh (\rho _{m})\pm b_{m}'( \pm 1)\sinh (\rho _{m}) \bigr]=0, \end{aligned}$$

66b $$\begin{aligned} &\sum_{m=1}^{N+1} \bigl[\rho _{m} b_{m}(1) \cosh (\rho _{m})+ \rho _{m} a_{m}(1) \sinh (\rho _{m}) \bigr]=0, \end{aligned}$$

66c $$\begin{aligned} &\sum_{m=1}^{N+1} \bigl[\rho _{m} b_{m}(-1) \cosh (\rho _{m})- \rho _{m} a_{m}(-1) \sinh (\rho _{m}) \bigr]=0. \end{aligned}$$

Note that equations (66b) and (66c) are equivalent to

67 $$ \begin{aligned} &\sum_{m=1}^{N+1}a_{m}(-1)S^{z,\mathrm{even}}_{N+1,m}- \sum_{m=1}^{N+1}b_{m}(-1)S^{z,\mathrm{odd}}_{N+1,m}=0, \\ &\sum_{m=1}^{N+1}a_{m}(1)S^{z,\mathrm{even}}_{N+1,m}+ \sum_{m=1}^{N+1}b_{m}(1)S^{z,\mathrm{odd}}_{N+1,m}=0. \end{aligned} $$

If we combine equations (67) with the terms in square brackets in (64c) and (64d), we get the matrix equations:

68 $$ \begin{aligned} &S^{z,\mathrm{even}}\mathbf{a}(-1)-S^{z,\mathrm{odd}} \mathbf{b}(-1)= \mathbf{0,} \\ &S^{z,\mathrm{even}}\mathbf{a}(1)+S^{z,\mathrm{odd}}\mathbf{b}(1)=\mathbf{0}. \end{aligned} $$

The term in square brackets in (64b) vanishes if the following two equations vanish:

69a $$\begin{aligned} &\frac{\partial }{\partial x}\sum_{m=1}^{N+1} \bigl[a_{m}'(x)\cosh ( \rho _{m} x)+b_{m}'(x)\sinh (\rho _{m} x) \bigr]=0, \end{aligned}$$

69b $$\begin{aligned} &\sum_{m=1}^{N+1} \bigl[\rho _{m} b_{m}'(x)\cosh (\rho _{m} x)+\rho _{m} a_{m}'(x) \sinh (\rho _{m} x) \bigr]=0. \end{aligned}$$

We see that in equation (69a) the sum should be constant. Using equation (66a), we see that this constant is zero.

70 $$ \sum_{m=1}^{N+1} \bigl[a_{m}'(x)\cosh (\rho _{m} x)+b_{m}'(x)\sinh ( \rho _{m} x) \bigr]=0. $$

The remaining equations (64e), (69b), (70) form a system of differential equations with boundary conditions (68):

71 $$\begin{aligned}& \sum_{m=1}^{N+1} \biggl[ \frac{a_{m}'(x)}{k_{j} ^{2}-\rho _{m}^{2}}k_{j} \cosh \bigl(\rho _{m} x'\bigr)+ \frac{b_{m}'(x)}{k_{j} ^{2}-\rho _{m}^{2}}k_{j} \sinh \bigl(\rho _{m} x'\bigr) \biggr]=0, \\& \begin{aligned}&\sum_{m=1}^{N+1} \biggl[ \frac{a_{m}'(x)}{k_{j} ^{2}-\rho _{m}^{2}} \rho _{m} \sinh \bigl(\rho _{m} x'\bigr)+ \frac{b_{m}'(x)}{k_{j} ^{2}-\rho _{m}^{2}}\rho _{m} \cosh \bigl(\rho _{m} x'\bigr) \biggr]=-R(z,B)y(x), \\ &\sum_{m=1}^{N+1} \bigl[\rho _{m} b_{m}'(x)\cosh (\rho _{m} x)+\rho _{m} a_{m}'(x) \sinh (\rho _{m} x) \bigr]=0,\end{aligned} \\& \sum_{m=1}^{N+1} \bigl[a_{m}'(x) \cosh (\rho _{m} x)+b_{m}'(x)\sinh ( \rho _{m} x) \bigr]=0. \end{aligned}$$

We can rewrite these equations by introducing some matrices. We define the diagonal matrices *Ĉ*, $\hat{S} \in C(\Omega ,\mathbb{C}^{(N+1)\times (N+1)})$, the square matrices *K̂*, *M̂*, $\hat{Q} \in \mathbb{C}^{(N+1)\times (N+1)}$ and the operator $\hat{R} : Y\rightarrow Y^{N+1}$ as follows:

72 $$ \begin{aligned} &\hat{C}_{m,m}(x)=\cosh (\rho _{m} x), \\ &\hat{S}_{m,m}(x)=\sinh (\rho _{m} x), \\ &\hat{K}_{j,m}=\rho _{m} \hat{Q}_{j,m}, \\ &\hat{M}_{j,m}=k_{j} \hat{Q}_{j,m}, \\ &\hat{Q}_{j,m}= \textstyle\begin{cases} \frac{1}{k_{j} ^{2}-\rho _{m}^{2}} & \text{for } j\in \{1,\ldots ,N \}, \\ 1 & \text{for } j=N+1, \end{cases}\displaystyle \\ &(\hat{R}y)_{i}= \textstyle\begin{cases} R(z,B)y & \text{for } j\in \{1,\ldots ,N\}, \\ 0 & \text{for } j=N+1. \end{cases}\displaystyle \end{aligned} $$

Here $j,m \in \{1,\ldots ,N+1\}$, and we define $k_{N+1}:=1$.

We seek functions $\mathbf{a}(x)$ and $\mathbf{b}(x)$ which solve the system of differential equations

73 $$ \begin{aligned} &\hat{M}\bigl(\hat{C}(x)\mathbf{a}'(x)+ \hat{S}(x)\mathbf{b}'(x)\bigr)= \mathbf{0,} \\ &\hat{K}\bigl(\hat{S}(x)\mathbf{a}'(x)+\hat{C}(x) \mathbf{b}'(x)\bigr)=-\hat{R}y(x), \end{aligned} $$

with boundary conditions

74 $$ \begin{aligned} &S^{z,\mathrm{even}}\mathbf{a}(-1)-S^{z,\mathrm{odd}} \mathbf{b}(-1)= \mathbf{0,} \\ &S^{z,\mathrm{even}}\mathbf{a}(1)+S^{z,\mathrm{odd}}\mathbf{b}(1)=\mathbf{0}. \end{aligned} $$

For $z\in \rho (A)$, we have that $S^{z,\mathrm{odd}}$ and $S^{z,\mathrm{even}}$ are invertible. Due to Lemmas 34 and 35, when $z\notin \mathcal{S}$, *Q̂* satisfies the conditions of Lemma 47, and hence *Q̂* is invertible. We can write the determinant of *K̂* and *M̂* in terms of the determinant of *Q̂*, $\det (\hat{M})=\det (\hat{Q})\prod_{j=1}^{N} k_{j} $, $|\hat{K}|=\det (\hat{Q})\prod_{m=1}^{N+1}\rho _{m}$, and so *K̂* and *M̂* are both invertible too.

Now we multiply the first line of (73) by $\hat{C}(x)\hat{M}^{-1}$ and the second line by $\hat{S}(x)\hat{K}^{-1}$

75 $$ \begin{aligned} &\hat{C}^{2}(x)\mathbf{a}'(x)+ \hat{C}(x)\hat{S}(x) \mathbf{b}'(x)=\mathbf{0}, \\ &\hat{S}^{2}(x)\mathbf{a}'(x)+\hat{C}(x)\hat{S}(x) \mathbf{b}'(x)=- \hat{S}(x)\hat{K}^{-1}\hat{R}y(x). \end{aligned} $$

If we now subtract these equations and use the trigonometric identity $\hat{C}^{2}(x)-\hat{S}^{2}(x)=I$, we arrive at the following equation:

76 $$ \begin{aligned} &\mathbf{a}'(x)=\hat{S}(x) \hat{K}^{-1}\hat{R}y(x), \\ &\mathbf{b}'(x)=-\hat{C}(x)\hat{K}^{-1}\hat{R}y(x). \end{aligned} $$

Here, we get the second line by a similar procedure. We note that $\hat{R}y \in C^{2}(\Omega )$ and $A(x),B(x) \in C^{\infty }(\Omega )$, which implies that $\mathbf{a}(x), \mathbf{b}(x) \in C^{3}(\Omega )$. Hence we satisfy the regularity condition.

We can now find $\mathbf{a}(x)$ and $\mathbf{b}(x)$ by taking an anti-derivative plus some constants of integration $\mathbf{a}^{c}$ and $\mathbf{b}^{c}$. To satisfy the boundary equations (74), we take an anti-derivative such that $\mathbf{a}(-1)+\mathbf{a}(1)=2\mathbf{a}^{c}$ and $\mathbf{b}(-1)+\mathbf{b}(1)=2\mathbf{b}^{c}$.

77 $$ \begin{aligned} &\mathbf{a}(x)=\mathbf{a}^{c}+ \frac{1}{2} \biggl( \int _{-1}^{x} \hat{S}\bigl(x' \bigr)\hat{K}^{-1} \hat{R}y\bigl(x'\bigr)\,dx'- \int _{x}^{1} \hat{S}\bigl(x' \bigr) \hat{K}^{-1} \hat{R}y\bigl(x'\bigr)\,dx' \biggr), \\ &\mathbf{b}(x)=\mathbf{b}^{c}-\frac{1}{2} \biggl( \int _{-1}^{x} \hat{C}\bigl(x' \bigr)\hat{K}^{-1} \hat{R}y\bigl(x'\bigr)\,dx'- \int _{x}^{1} \hat{C}\bigl(x' \bigr) \hat{K}^{-1} \hat{R}y\bigl(x'\bigr)\,dx' \biggr). \end{aligned} $$

By adding and subtracting boundary equations (74), we find that the constants of integration equal

78 $$ \begin{aligned} &\mathbf{a}^{c}= \frac{1}{2} \bigl(S^{z,\mathrm{even}}\bigr)^{-1}S^{z,\mathrm{odd}} \biggl( \int _{-1}^{1} \hat{C}\bigl(x' \bigr)\hat{K}^{-1} \hat{R}y\bigl(x'\bigr)\,dx' \biggr), \\ &\mathbf{b}^{c}=-\frac{1}{2}\bigl(S^{z,\mathrm{odd}} \bigr)^{-1}S^{z,\mathrm{even}} \biggl( \int _{-1}^{1} \hat{S}\bigl(x' \bigr)\hat{K}^{-1} \hat{R}y\bigl(x'\bigr)\,dx' \biggr). \end{aligned} $$

We can simplify this as follows:

79 $$ \begin{aligned} &\mathbf{a}(x)= \frac{1}{2} \int _{-1}^{1} \bigl( \hat{S} \bigl(x'\bigr)\mathrm{sgn}\bigl(x-x'\bigr)+ \bigl(S^{z,\mathrm{even}}\bigr)^{-1}S^{z,\mathrm{odd}}\hat{C} \bigl(x'\bigr) \bigr)\hat{K}^{-1} \hat{R}y \bigl(x'\bigr)\,dx', \\ &\mathbf{b}(x)=-\frac{1}{2} \int _{-1}^{1} \bigl(\hat{C}\bigl(x' \bigr) \mathrm{sgn}\bigl(x-x'\bigr)+\bigl(S^{z,\mathrm{odd}} \bigr)^{-1}S^{z,\mathrm{even}}\hat{S}\bigl(x'\bigr) \bigr) \hat{K}^{-1} \hat{R}y\bigl(x'\bigr)\,dx'. \end{aligned} $$

 □

For the computation of the first Lyapunov coefficient $l_{1}$, we need to evaluate the Dunford integral in (47). Similar to Dijkstra et al. [34], we can use residue calculus to find an expression for this integral.

#### Theorem 38

*Let* $\lambda \in \sigma _{p}(A)$ *be a simple eigenvalue and* $\lambda \notin \mathcal{S}$. *Let* $C_{\lambda }$ *be a sufficiently small closed disk such that* $C_{\lambda }\cap \sigma (A)=\{\lambda \}$ *and* $C_{\lambda } \cap \mathcal{S}=\emptyset $.

*If* *λ* *is an ‘even’ eigenvalue with eigenvector*

80 $$ \psi (\theta ) (x)= e^{\lambda \theta }\sum_{m=1}^{N+1} a_{m} \cosh \bigl( \rho _{m}(\lambda ) x\bigr), $$

*where* **a** *is a nontrivial solution of* $S^{\lambda ,\mathrm{even}}\mathbf{a}=0$, *then*

81 $$ \frac{1}{2\pi i} \oint _{\partial C_{\lambda }} e^{z \theta } \Delta ^{-1}(z)y \,dz= \nu \psi (\theta ) $$

*if and only if*

82 $$ \frac{\mathrm{adj}(S^{\lambda ,\mathrm{even}})}{2 \frac{d}{dz}(\det (S^{\lambda ,\mathrm{even}}))|_{z=\lambda }}S^{ \lambda ,\mathrm{odd}} \int _{-1}^{1}\hat{C}\bigl(x' \bigr)\hat{K}^{-1} \hat{R}y\bigl(x'\bigr)\,dx'= \nu \mathbf{a} $$

*for all* $y\in Y$, *where* $\mathrm{adj}(S^{\lambda ,\mathrm{even}})$ *denotes the adjugate of* $S^{\lambda ,\mathrm{even}}$, *and using the definitions in* (72).

*If* *λ* *is an ‘odd’ eigenvalue with eigenvector*

83 $$ \psi (\theta ) (x)= e^{\lambda \theta } \sum_{m=1}^{N+1} b_{m} \sinh \bigl( \rho _{m}(\lambda ) x\bigr), $$

*where* **b** *is a nontrivial solution of* $S^{\lambda ,\mathrm{odd}}\mathbf{b}=0$, *then*

84 $$ \frac{1}{2\pi i} \oint _{\partial C_{\lambda }} e^{z \theta } \Delta ^{-1}(z)y \,dz= \nu \psi (\theta ) $$

*if and only if*

85 $$ \frac{-\mathrm{adj}(S^{\lambda ,\mathrm{odd}})}{2 \frac{d}{dz}(\det (S^{z,\mathrm{odd}}))|_{z=\lambda }}S^{ \lambda ,\mathrm{even}} \int _{-1}^{1}\hat{B}\bigl(x' \bigr)\hat{K}^{-1} \hat{R}y\bigl(x'\bigr)\,dx'= \nu \mathbf{b} $$

*for all* $y\in Y$, *where* $\mathrm{adj}(S^{\lambda ,\mathrm{odd}})$ *denotes the adjugate of* $S^{\lambda ,\mathrm{odd}}$, *and using the definitions in* (72).

#### Proof

As $\sigma _{p}(A)$ and $\sigma _{p}(B)$ contain only isolated eigenvalues and $\rho _{m}(z)$ and $\det (P^{z}(k_{i,j}(z)))$ are analytic in *z*, the set $\mathcal{S}$ contains only isolated values. Hence such $C_{\lambda }$ exists.

Suppose that *λ* is an even eigenvalue. As $\mathcal{S}\cap C_{\lambda }=\emptyset $ and $\sigma (A)\cap C_{\lambda }=\{\lambda \}$, we have that $\Delta ^{-1}(z)\mathbf{y}$ is given by Theorem 37 for $z\in C_{\lambda }$. We observe that all components of the resolvent are analytic for all $z\in C_{\lambda }$ except for the constants of integration $\mathbf{a}^{c}(z)$. This analyticity simplifies (81) to

$$ \frac{e^{\lambda \theta }}{2\pi i} \sum_{m=1}^{N(N+1)} \cosh \bigl(\rho _{m}( \lambda ) x\bigr) \oint _{\partial C_{\lambda }} a_{m}^{c}(z)\,dz= \nu e^{ \lambda \theta } \sum_{m=1}^{N(N+1)} a_{m} \cosh \bigl(\rho _{m}(\lambda ) x\bigr) $$

for all $x\in \Omega $, $\theta \in [-h,0]$. We can substitute (78) and use the residue formula

$$ \frac{1}{2\pi i} \oint _{\partial C_{\lambda }} \bigl(S^{z,\mathrm{even}}\bigr)^{-1}\,dz = \mathrm{Res} \biggl(\frac{\mathrm{adj}(S^{z,\mathrm{even}})}{\det (S^{z,\mathrm{even}})}, \lambda \biggr)= \frac{\mathrm{adj}(S^{\lambda ,\mathrm{even}})}{\frac{d}{dz}(\det (S^{z,\mathrm{even}}))|_{z=\lambda }}. $$

Due to linear independence of $\cosh (\rho _{m}(\lambda ) x)$ for $m \in \{1,\ldots , N+1\}$, this results in the formula

$$ \frac{\mathrm{adj}(S^{\lambda ,\mathrm{even}})}{2 \frac{d}{dz}(\det (S^{z,\mathrm{even}}))|_{z=\lambda }}S^{ \lambda ,\mathrm{odd}} \int _{-1}^{1}\hat{C}\bigl(x' \bigr)\hat{K}^{-1} \hat{R}y\bigl(x'\bigr)\,dx'= \nu \mathbf{a}. $$

The reasoning for odd eigenvalues is similar. □

## Numerical results

In this section we examine a specific numerical example. We compute eigenvalues and the first Lyapunov coefficient for a Hopf bifurcation and investigate the effect of varying the diffusion parameter *d*.

For *J*, we choose the following difference of two exponentials, as in [34]:

86 $$ J\bigl(x,x'\bigr)= \frac{25}{2}e^{-2|x-x'|}-10 e^{-|x-x'|}. $$

**Figure 2:**
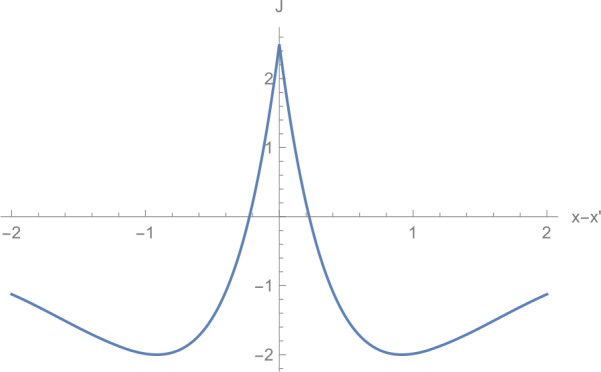
The wizard-hat connectivity of (86)

This connectivity is a model of a population of excitatory neurons acting on a short distance combined with a population of inhibitory neurons acting on a longer distance, see Fig. 2.

For the activation function *S*, we choose the sigmoidal function

87 $$ S(u)=\frac{1}{1+e^{-\gamma u}}-\frac{1}{2}. $$

As *S* is an odd function, $S''(0)=0$ and hence $D^{2}G(0) \equiv 0$. This simplifies the computation of first Lyapunov coefficient $l_{1}$ of (47) to

88 $$ \frac{1}{4\pi i} \oint _{\partial C_{\lambda }} e^{z\theta } \Delta ^{-1}(z)D^{3}G(0) ( \psi ,\psi ,\bar{\psi })\,dz= c_{1} \psi (\theta ). $$

We can compute this integral using Theorem 38 with $y= \frac{1}{2}D^{3}G(0)(\psi ,\psi ,\bar{\psi })$.

We fix the following values for parameters $\alpha =1$ and $\tau ^{0}=\frac{3}{4}$ and use *γ* as the bifurcation parameter. We want to compare two cases: without diffusion, i.e. $d=0$, and with diffusion, i.e. $d>0$.

### Hopf bifurcation

For $d=0$, we have a Hopf bifurcation for $\gamma =3.3482$ at $\lambda =1.2403i$ with the corresponding eigenvector

89 $$ \begin{aligned} \psi (\theta ) (x)={}&e^{1.2403 i \theta } \bigl[0.9998\cosh \bigl((0.2770-0.8878i)x\bigr) \\ &{}\times (-0.0178+0.0050i)\cosh \bigl((3.7185+3.2284i)x\bigr)\bigr]. \end{aligned} $$

The normal form coefficient $c_{1}=-1.132-0.282i$ and the Lyapunov coefficient $\ell _{1}=-0.9123$, and hence the bifurcation is supercritical.

For $d=0.2$, we have a Hopf bifurcation for $\gamma =3.3094$ at $\lambda =1.2379i$ with the corresponding eigenvector

90 $$ \begin{aligned} \psi (\theta ) (x)={}&e^{1.2379 i \theta } \bigl[0.9972\cosh \bigl((0.2535-0.8490i)x\bigr) \\ &{}+(-0.0727-0.0177i)\cosh \bigl((1.7315+3.2475i)x\bigr) \\ &{}+(0.0029-0.0060i)\cosh \bigl((3.90746+0.3586i)x\bigr)\bigr]. \end{aligned} $$

The normal form coefficient $c_{1}=-1.153-0.258i$ and the Lyapunov coefficient $\ell _{1}=-0.9314$, and hence the bifurcation is also supercritical. We have put these values for further reference in Table 1.

**Table 1:** Parameter values of the Hopf bifurcation without and with diffusion respectively

Bifurcation	*α*	$\tau ^{0}$	$\eta _{1}$	$\eta _{2}$	$\mu _{1}$	$\mu _{2}$	*d*	*γ*	*λ*	$\ell _{1}$
Hopf 1	1	0.75	12.5	−10	2	1	0	3.3482	1.2403i	−0.9123
Hopf 2	1	0.75	12.5	−10	2	1	0.2	3.3094	1.2379i	−0.9314

**Figure 3:**
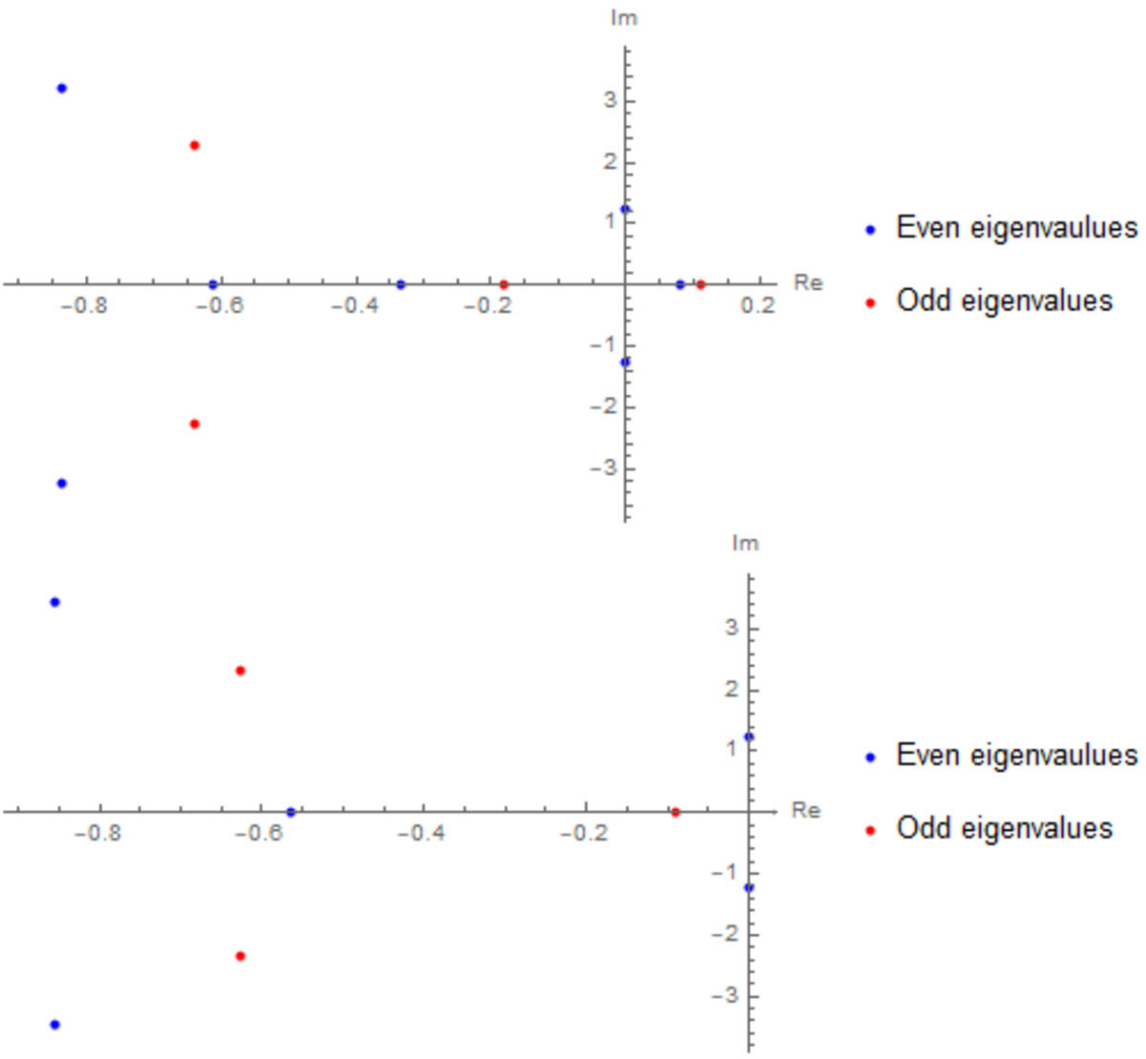
The eigenvalues of *A* at parameter values in Table 1 of the Hopf bifurcation without and with diffusion respectively

**Figure 4:**
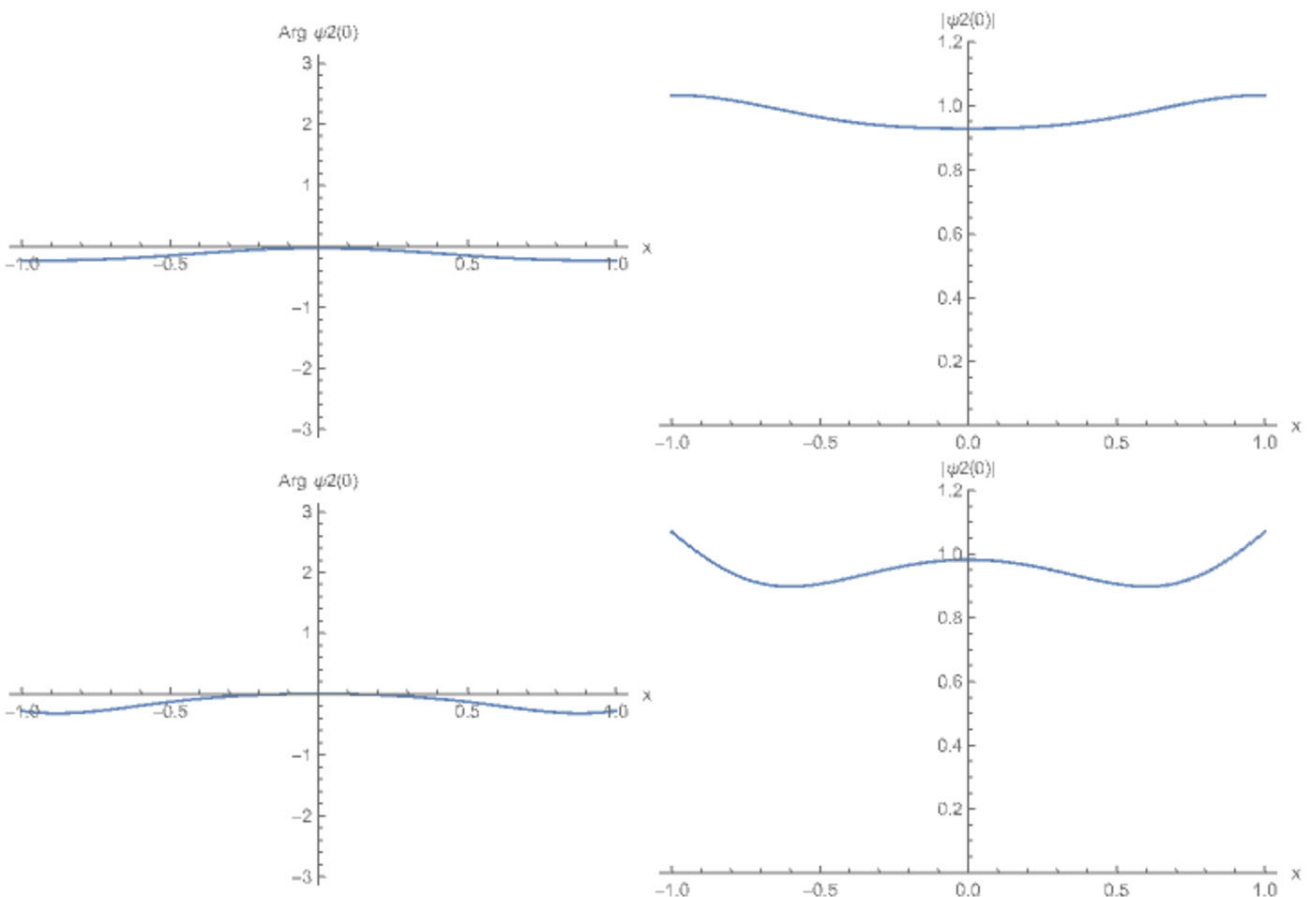
The corresponding eigenvectors of the eigenvalue $\lambda = \omega i$ at parameter values in Table 1 without and with diffusion respectively. Note that with diffusion the eigenvector satisfies the boundary conditions at $x=1$ and $x=-1$, while this is not the case without diffusion

As one might already have observed, the diffusion has little effect on the Hopf bifurcation. We observe more generally that the eigenvalues which are off the real axis are barely effected by the introduction of diffusion, while the eigenvalues on the real axis become more negative, see Fig. 3.^1^ A possible explanation is that the eigenvector corresponding to the eigenvalue on the imaginary axis has very little spatial curvature, see Fig. 4. As diffusion penalises curvature, its effect on this eigenvector would be small.

### Discretisation

To obtain an approximate solution of (ADDE), we discretise the spatial domain Ω into an equidistant grid of $n^{x}$ points, $x_{1}, \dots , x_{n^{x}}$, with a width of $\delta = \frac{2}{n^{x}-1}$. As in [29], we discretise the integral operator *G* using the trapezoidal rule and the diffusion operator *B* using a central difference method and a reflection across the boundary for the boundary conditions. This results in a second order spatial discretisation. The discretisation of (ADDE) for $n \in \{1,\ldots , n^{x}\}$ and $t\in \mathbb{R}^{+}$ becomes a set of delay equations (DDE):

DDE $$ \textstyle\begin{cases} \frac{\partial u}{\partial t}(t,x_{n}) \\ \quad = \frac{d}{2\delta ^{2}} (u(t,x_{n-1}) - 2 u(t,x_{n}) + u(t,x_{n+1})) - \alpha u(t,x_{n}) \\ \qquad {}+ \delta \sum_{m=1}^{n^{x}} \xi _{m} J(x_{n},x_{m})S(u(t-\tau (x_{n},x_{m}),x_{m})), \\ u(t,x_{0})= u(t,x_{2}), \\ u(t,x_{n^{x}+1})= u(t,x_{n^{x}-1}), \\ u(t,x_{n})=\varphi (t,x_{n}). \end{cases} $$

Here $\xi _{m}$ is defined as

91 $$ \xi _{m}= \textstyle\begin{cases} 1 & m \in \{2, \ldots , n^{x}-1\}, \\ \frac{1}{2}& m=1 \text{or } m=n^{x}. \end{cases} $$

Now we are left with a set of $n^{x}$ ordinary delay differential equations which we solve with a standard DDE-solver. Note that (DDE) is very similar to the discrete model (3) from which (ADDE) is derived. Only the terms at the boundary are different due to the second order discretisation.

### Simulations

We will now perform some simulations around the Hopf bifurcation with diffusion. We set $n^{x}=50$ and take as initial conditions an odd function and an even function:

92 $$ \begin{aligned} &\varphi _{1}(\theta ) (x)= \frac{1}{5}\sin {\frac{1}{2} \pi x}, \\ &\varphi _{2}(\theta ) (x)= \frac{1}{5}\cos {\pi x}. \end{aligned} $$

**Figure 5:**
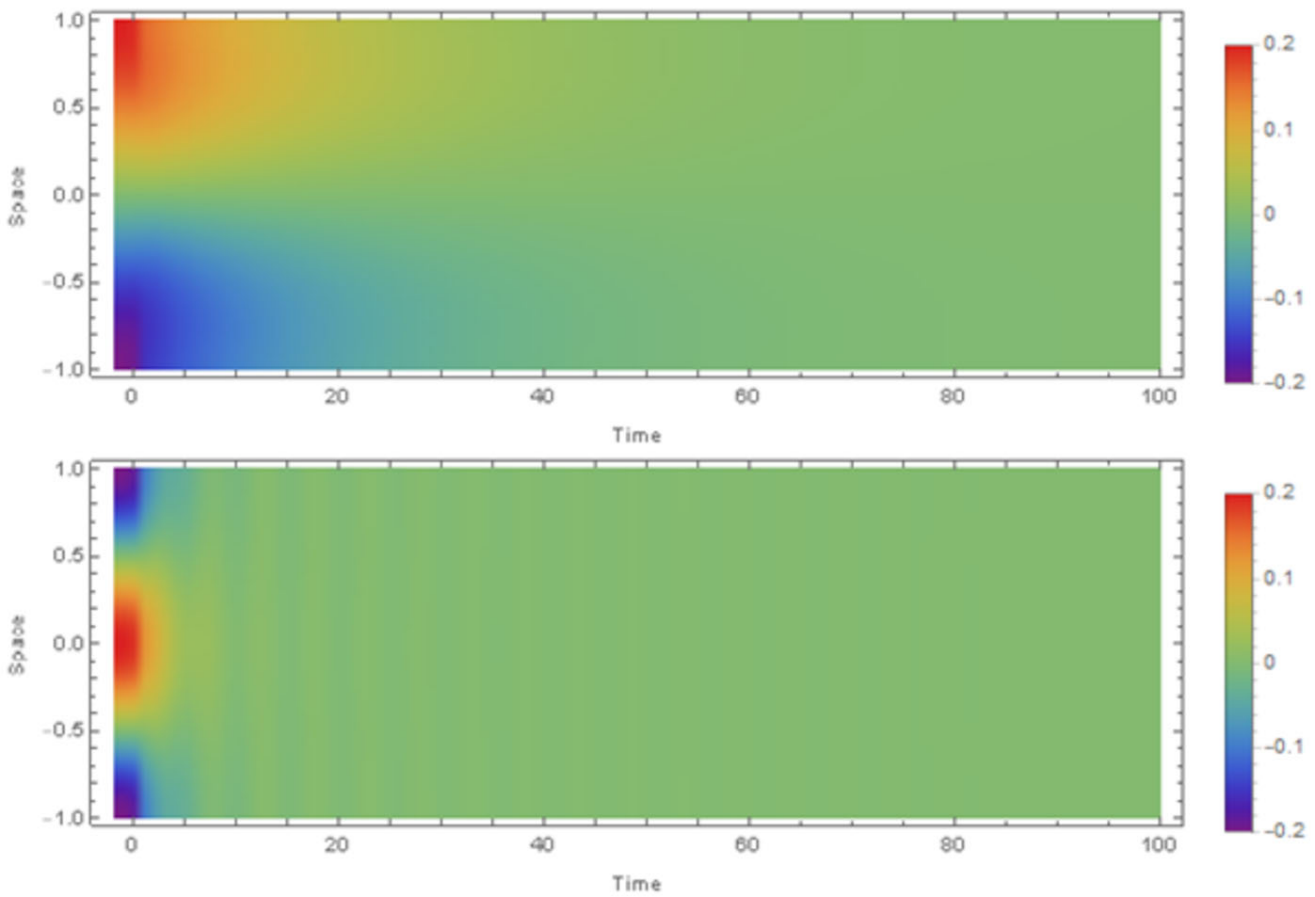
Simulation of (DDE) with the initial conditions $\varphi _{1}$, $\varphi _{2}$ of (92) and $\gamma = 3$ and $d=0.2$

**Figure 6:**
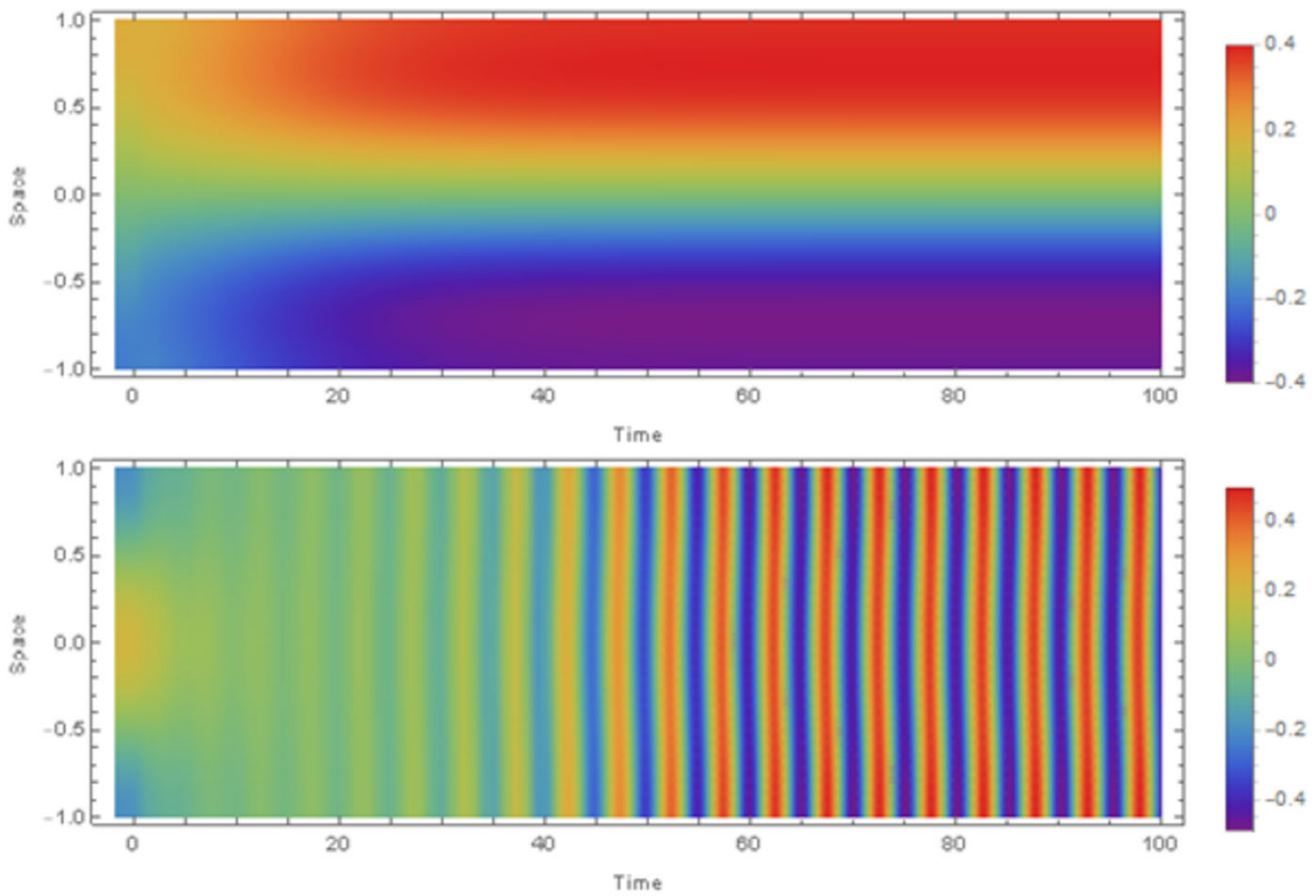
Simulation of (DDE) with the initial conditions $\varphi _{1}$, $\varphi _{2}$ of (92) and $\gamma = 4$ and $d=0.2$

For Fig. 5, we took $\gamma =3$, and for Fig. 6, $\gamma =4$.

**Figure 7:**
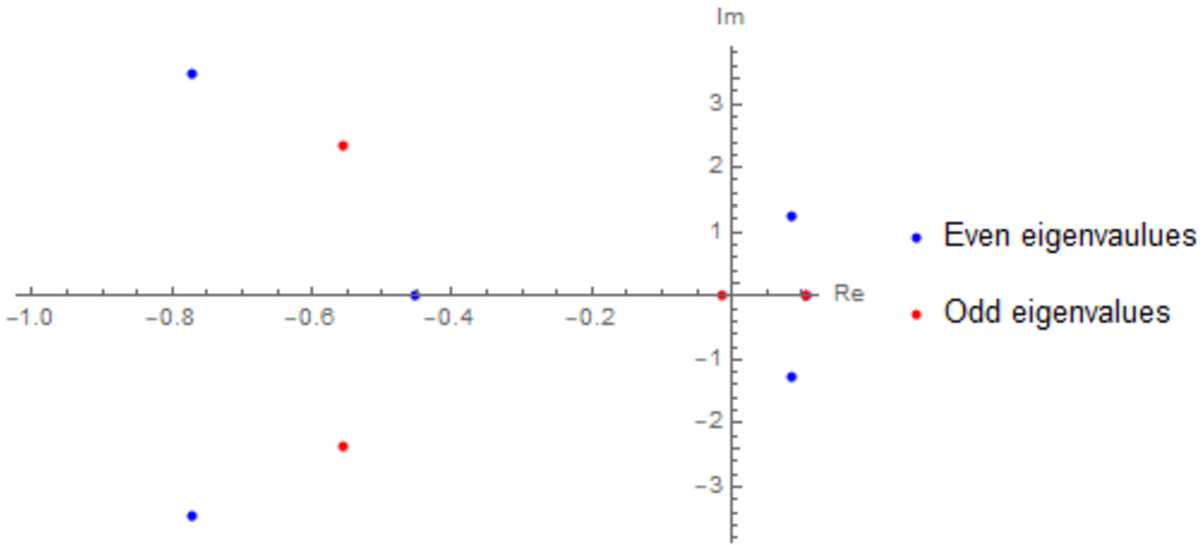
The eigenvalues of *A* for $\gamma = 4$ and $d=0.2$

For $\gamma =3$, the solutions with both initial conditions (92) converge to the trivial equilibrium. The one with the odd initial condition converges monotonously to the trivial equilibrium, while the one with the even initial condition converges to the trivial equilibrium in an oscillatory manner. For $\gamma =4$, there are (at least) two nontrivial stable states. The odd initial condition converges to some nontrivial equilibrium, and the even initial condition converges to some limit cycle, which is due to the Hopf bifurcation. This is similar to the results of Dijkstra et al. [34], where the nontrivial equilibrium arises from a pitchfork bifurcation. The bi-stability is also exemplified in the eigenvalues, see Fig. 7, as we have a positive real eigenvalue and a pair of complex eigenvalues with a positive real component.

**Figure 8:**
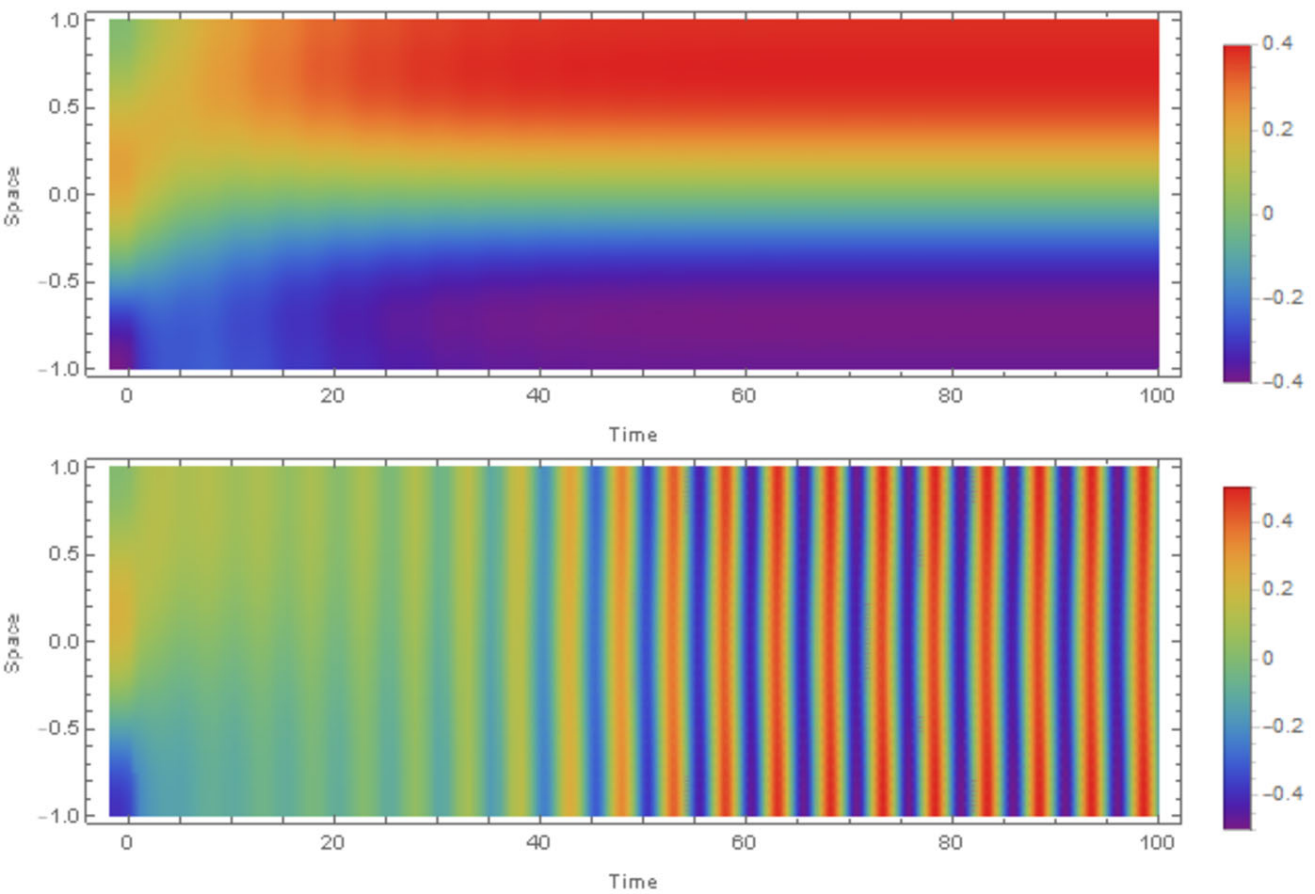
Simulation of (DDE) with the same initial condition $\varphi _{3}$ (93) and $\gamma = 4$, $d=0.2$ and $\gamma = 4$, $d=0.5$ respectively

We have seen that increasing the value of *d* decreases the eigenvalues on the real axis. This would imply that the nontrivial equilibrium becomes unstable or disappears, probably through a pitchfork bifurcation. Indeed when we use the initial condition

93 $$ \varphi _{3} = \varphi _{1} + \varphi _{2} $$

and compare the dynamics for $d=0.2$ and $d=0.5$ in Fig. 8. The initial condition converges to a nontrivial equilibrium when $d=0.2$, but it converges to a limit cycle when $d=0.5$.

## Discussion

We have proved the necessary theorems to construct the sun-star calculus for abstract delay differential equations. In particular, we proved a novel characterisation for sun-reflexivity in Theorem 12. The sun-star calculus provides a variation-of-constants formulation for the nonlinear problem and produces results on the spectral properties of the system, notably the essential spectrum. Using the results of Janssens [6] on the centre manifold reduction, we have derived a simple and explicit formula to compute the first Lyapunov coefficient for the Hopf bifurcation. This procedure can quite easily be extended to normal coefficients of other local bifurcations.

The neural field models, both with and without diffusion, can be cast as abstract delay differential equations to which the same theoretical results can be applied. In the sun-star calculus the relevant spaces, duality pairings and Fredholm alternative follow naturally by considering the strong continuity of adjoint operators. Hence there is no need to construct formal projectors. Moreover, for a specific example of the neural field, we could calculate the first Lyapunov coefficient exactly and with arbitrary precision. Thus we conclude that the sun-star calculus for delay equations is a natural setting to study neural field models, with and without diffusion.

For certain specific connectivity functions, we have derived analytical conditions for *λ* to be an eigenvalue for a neural field with a connectivity function that is a sum of exponentials. We have also constructed the corresponding eigenvectors and the resolvent. Numerical results show that the diffusion term does not cause oscillations to arise due to a Hopf bifurcation. However, stable equilibria which are not uniform disappear due to the smoothing effect of the diffusion. So increasing the diffusion in a bi-stable system with a nonuniform equilibrium and a synchronous oscillation leads to a system with only stable synchronous oscillations. We hypothesise that this is a more general feature of equations with diffusion and a delayed reaction.

Gap junctions, modelled by the diffusion term in our neural field, are thought to be linked to synchronisation in Parkinson’s disease [3]. Further research could be undertaken to see whether the effects can be observed in a neural field model with physiological values for the parameters.

We used a neural field model with a connectivity function, which is a sum of exponentials. This connectivity function is commonly used to aggregate the effect of multiple different types of cells, e.g. excitatory and inhibitory neurons. However, introducing a diffusion term into this model leads to gap junctions between similar and different populations of neurons of the same strength. This may not be physiologically feasible. A way to circumvent this is to use a neural field model with multiple populations. In such a model, it is possible to introduce only gap junctions between neurons of the same population.

We have studied a neural field on a one-dimensional closed domain. However, when modelling the neuronal activity in the cortex, it is common to use two-dimensional domains [24]. For a neural field with a rectangular domain, characterising the spectrum, as is done in this paper in Sect. 3, is still an open problem. On a spherical domain, Visser et al. [35] have characterised the spectrum for a neural field with transmission delays and have computed normal form coefficients of Hopf and double Hopf bifurcations. It seems possible to extend the analysis of that paper to include a diffusion term into that neural field model. Due to the general nature of the theoretical results of Sect. 2, these results, including the sun-star framework, the variation of constants formulation and the essential spectrum, also hold for neural field models on arbitrary domains.

## Data Availability

Not applicable.

## Appendix A: Properties of the diffusion operator

In this appendix we investigate the properties of the diffusion operator *B* in the context of the sun-star calculus. We consider the space of continuous functions $Y=C(\Omega )$, where we take our domain Ω to be the interval $[-1,1]$. We define $B: D(B) \rightarrow Y$, an unbounded, closed, linear operator as follows:

94 $$ \begin{aligned} &B q:= d q'' - \alpha q, \\ &D(B):= \bigl\{ q\in Y | q\in C^{2}(\Omega ), q'( \partial \Omega )=0 \bigr\} . \end{aligned} $$

### A.1 Spectral properties

We start our analysis with this result on the semigroup *S* generated by *B*.

#### Lemma 39

([58, Proposition VI.6.19])

*The operator* $(B,D(B))$ *generates a strongly continuous*, *positive and immediately compact semigroup* $(S(t))_{t\geq 0}$.

Sturm–Liouville theory gives the following well-known results on the spectral properties of the diffusion operator. We can explicitly derive the eigenvalues and eigenvectors using separation of variables. This is entirely standard and therefore the calculation is omitted.

#### Lemma 40

*For the spectrum of* *B*, *we have that* $\sigma (B)=\sigma _{p}(B)$. *All eigenvalues of* *B* *are simple and given by* $\lambda _{n}^{\mathrm{even}}=-dn^{2}\pi ^{2}-\alpha $ *with even eigenvector* $\cos (n\pi x)$ *and* $\lambda _{n}^{\mathrm{odd}}=-d(n+\frac{1}{2})^{2}\pi ^{2}-\alpha $ *with odd eigenvector* $\sin ((n+\frac{1}{2})\pi x)$ *for all* $n\in \mathbb{N}_{0}$. *Moreover*, *these eigenvectors form a maximal set in* *Y*, *i*.*e*. *their span is dense in* *Y*.

We can also explicitly find an explicit representation of the semigroup $S(t)$ and resolvent $R(z,B)$ in terms of the eigenvectors.

#### Lemma 41

*The semigroup* *S* *can be explicitly written as a convolution*

95 $$ S(t)\varphi (x)= \int _{\Omega }\varphi \bigl(x'\bigr)G \bigl(t,x,x'\bigr)\,dx' $$

*with Green’s function*

96 $$ \begin{aligned} G\bigl(t,x,x'\bigr):={}&\sum _{n=0}^{\infty } \biggl((1+\delta _{0n})^{-1} \cos (n\pi x)\cos \bigl(n\pi x'\bigr)e^{ (-dn^{2}\pi ^{2}-\alpha )t} \\ &{} +\sin \biggl(\biggl(n+\frac{1}{2}\biggr)\pi x\biggr)\sin \biggl( \biggl(n+ \frac{1}{2}\biggr)\pi x'\biggr)e^{ (-d (n+\frac{1}{2} )^{2}\pi ^{2}- \alpha )t} \biggr). \end{aligned} $$

*The resolvent* $R(z,B): Y \rightarrow D(B)$ *for* $z \in \rho (B)$ *can be explicitly written as a convolution*

97 $$ R(z,B)y(x)= \int _{\Omega }y\bigl(x'\bigr)G^{z} \bigl(x,x'\bigr)\,dx' $$

*with Green’s function*

98 $$ \begin{aligned} &G^{z}\bigl(x,x'\bigr) \\ &\quad := \sum_{n=0}^{\infty } \biggl( (1+\delta _{0n})^{-1} \bigl(z+\alpha +dn^{2}\pi ^{2} \bigr)^{-1}\cos (n\pi x)\cos \bigl(n\pi x'\bigr) \\ &\qquad {} +\biggl(z+\alpha +d \biggl(n+\frac{1}{2}\biggr)^{2}\pi ^{2} \biggr)^{-1}\sin \biggl(\biggl(n+ \frac{1}{2}\biggr)\pi x\biggr)\sin \biggl(\biggl(n+\frac{1}{2} \biggr)\pi x'\biggr) \biggr). \end{aligned} $$

*Here*, $\delta _{mn}$ *is the Kronecker delta*.

### A.2 Sun-star calculus

We will now develop the sun-star calculus for the diffusion operator *B*. We can take $d=1$ and $\alpha =0$ for this section, without loss of generality, as the sun-star calculus is invariant with respect to bounded perturbations of the generator of the semi-group.

As a consequence of the Riesz representation theorem, $Y^{*}$ can be represented as $\operatorname{NBV}(\Omega )$, the functions of bounded variation, normalised such that for $y\in Y^{*}$, $y(-1)=0$. The corresponding norm on $\operatorname{NBV}(\Omega )$ is the total variation norm, and the duality pairing is given by the Riemann–Stieltjes integral:

99 $$ \bigl\langle y^{*},y \bigr\rangle := \int _{-1}^{1} y \,dy^{*} $$

We will now try to find a representation for $B^{*}$.

#### Theorem 42

*The dual space* $Y^{*}$ *can be represented as* $\operatorname{NBV}(\Omega )$. *Furthermore*, $y^{*}\in D(B^{*})$ *if and only if for* $x\in (-1,1]$

100 $$ y^{*}(x)= c_{1} + \int _{-1}^{x} \biggl(c_{2} + \int _{-1}^{s} z^{*} \bigl(x'\bigr)\,dx' \biggr)\,ds, $$

*where* $c_{1},c_{2} \in \mathbb{R}$ *and* $z^{*} \in \operatorname{NBV}(\Omega )$ *with* $z^{*}(1)=0$. *For such* $y^{*}$, *we have that* $B^{*} y^{*}= z^{*}$.

#### Proof

We start by proving the ‘only if’ part of the theorem. Let $y^{*} \in D(B^{*})$, $y\in D(B)$ and $z^{*}=B^{*}y^{*}$. Furthermore, let

$$ w^{*}(s):=c_{2} + \int _{-1}^{s} z^{*} \bigl(x'\bigr)\,dx' $$

for some $c_{2} \in \mathbb{R}$. As $y\in C^{2}(\Omega )$ and $y'(\pm 1)=0$, we get that using integration by parts for Riemann–Stieltjes integrals [5, Proposition A.15, A.18, A.19]

$$\begin{aligned} \int _{-1}^{1}y''(x)\,dy^{*}(x) &= \bigl\langle y^{*}, B y\bigr\rangle \\ & = \bigl\langle z^{*}, y\bigr\rangle = \int _{-1}^{1}y \,dz^{*} \\ &= z^{*}(x)y(x)|_{-1}^{1} - \int _{-1}^{1}y'(x)z^{*}(x)\,dx \\ &= z^{*}(1)y(1)+ \int _{-1}^{1}y''(x)w^{*}(x)\,dx. \end{aligned}$$

If we take *y* as a constant function, then we immediately see that $z(1)=0$ is a necessary condition. For any $-1< x'< x<1$, we can take a sequence of $y_{n} \in D(B)$ such that $y_{n}''(s)$ converges monotone to the characteristic function on the interval $[x',x]$. Then, by the Lebesque monotone convergence theorem, we get that

$$ y^{*}(x)-y^{*}\bigl(x'\bigr)= \int _{x'}^{x}\,dy^{*}(s) = \int _{x'}^{x} w^{*}(s)\,ds. $$

Letting $x'\downarrow -1$, we get that

$$ y^{*}(x)= \lim_{x' \downarrow -1} y^{*} \bigl(x'\bigr) + \int _{-1}^{x} w^{*}(s)\,ds. $$

So we can write this $y^{*}$ as

$$ y^{*}(x)= c_{1} + \int _{-1}^{x} \biggl(c_{2} + \int _{-1}^{s} z^{*} \bigl(x'\bigr)\,dx' \biggr)\,ds. $$

Next we prove the ‘if’ part of the theorem. Let $y^{*}$ have the form in equation (100) with $z(1)=0$. Then, for all $y\in D(B)$, we have again by using integration by parts that

$$\begin{aligned} \bigl\langle y^{*}, B y\bigr\rangle &= \int _{-1}^{1}y''(x)\,dy^{*}(x) \\ &= \int _{-1}^{1}y''(x)w^{*}(x)\,dx \\ &= - \int _{-1}^{1}y'(x)z^{*}(x)\,dx \\ &= \int _{-1}^{1} y(x)\,dz^{*}(x) = \bigl\langle z^{*}, y\bigr\rangle . \end{aligned}$$

Hence we can conclude that $y^{*}\in D(B^{*})$ and $B^{*} y^{*} = z^{*}$. □

Now we are in a position to find $Y^{\odot }$, the sun-dual of *Y* with respect to *S*, which is the closure of $D(B^{*})$ with respect to the total variation norm.

#### Theorem 43

*The sun*-*dual* $Y^{\odot }$ *with respect to the semigroup* *S* *can be represented as* $\mathbb{R}\times L^{1}(\Omega )$. *For the sun*-*dual of* *B*, *we have that*

101 $$ D\bigl(B^{\odot }\bigr):=\bigl\{ \bigl(c,w^{\odot }\bigr)\in \mathbb{R}\times L^{1}(\Omega )| c \in \mathbb{R}, \bigl(w^{\odot }\bigr)'\in \operatorname{AC}\bigl([-1,1] \bigr) ,\bigl(w^{\odot }\bigr)'(1)=0\bigr\} $$

*and* $B^{\odot }(c,w^{\odot }) := ((w^{\odot })'(-1),(w^{\odot })'')$, *where* $(w^{\odot })''$ *is some* $L^{1}$ *function such that*

102 $$ \bigl(w^{\odot }\bigr)'(x)= \bigl(w^{\odot } \bigr)'(-1) + \int _{-1}^{x} \bigl(w^{\odot } \bigr)''(s)\,ds. $$

#### Proof

Let $y^{*} \in D(B^{*})$. Again using the notation we get that, for $x,s\in (-1,1]$,

$$\begin{aligned} &y^{*}(x)= c_{1} + \int _{-1}^{x} w^{*}(s)\,ds, \\ &w^{*}(s)=c_{2} + \int _{-1}^{s} z^{*} \bigl(x'\bigr)\,dx' \end{aligned}$$

for some $c_{1},c_{2} \in \mathbb{R}$ and $z^{*} \in \operatorname{NBV}(\Omega )$ with $z^{*}(1)=0$, we can rewrite the total variation norm as

$$ \bigl\Vert y^{*} \bigr\Vert _{Y^{*}}= \vert c_{1} \vert + \bigl\| w^{*} \bigr\| _{L^{1}}. $$

For the space

$$ W := \biggl\{ c + \int _{-1}^{s} z^{*} \bigl(x'\bigr)\,dx' \bigm| c \in \mathbb{R}, z^{*} \in \operatorname{NBV}(\Omega ), z^{*}(1)=0 \biggr\} , $$

we have that $\{w^{*} \in C^{2} | (w^{*})'(-1)=0\}\subset W \subset L^{1}$. As this first space of $C^{2}$ functions is dense in $L^{1}$, we have that *W* is dense in $L^{1}$. Hence, we can represent $Y^{\odot }$ as the space

$$ \biggl\lbrace y^{\odot }\in \operatorname{NBV}(\Omega )\bigm| y^{\odot }(x) = c + \int _{-1}^{x} w^{\odot }(s)\,ds \textit{ where } c\in \mathbb{R}, w^{\odot }\in L^{1}(\Omega ) \textit{ for } x \in (-1,1] \biggr\rbrace $$

which are the absolutely continuous functions on $(-1,1]$ with a jump from 0 to *c* at $x=-1$.

We can equivalently express $Y^{\odot }$ as $\mathbb{R}\times L^{1}(\Omega )$ where $y^{\odot }=(c,w^{\odot })$ with $c\in \mathbb{R}$ and $w^{\odot }\in L^{1}(\Omega )$ equipped with the norm

$$ \bigl\Vert y^{\odot } \bigr\Vert _{Y^{\odot }}:= \vert c_{1} \vert + \bigl\| w^{\odot } \bigr\| _{L^{1}}. $$

The domain of $B^{\odot }$ is defined as $D(B^{\odot })=\{y^{\odot }\in D(B^{*}) | B^{*} y^{\odot }\in Y^{\odot }\}$. Using equation (100) we have $B^{*}y^{*} = z^{*}$. If $z^{*} \in Y^{\odot }$, then $z^{*}$ must be absolutely continuous on $(-1,1]$. So for $y^{\odot }=(c,w^{\odot })$ we find that $(w^{\odot })'=z^{*}$ is absolutely continuous on $(-1,1]$. As $(w^{\odot })'$ is an $L^{1}$-function, we can redefine $(w^{\odot })'(-1):= (w^{\odot })'(-1+)$ to get an absolutely continuous function on $[-1,1]$. The boundary condition $z(1)=0$ is transformed into $(w^{\odot })'(1)=0$

Thus we can write that $B^{\odot }(c,w^{\odot })= ((w^{\odot })'(-1), (w^{\odot })'')$, where $(w^{\odot })''$ is an $L^{1}$ function such that

$$ \bigl(w^{\odot }\bigr)'(x)= \bigl(w^{\odot } \bigr)'(-1) + \int _{-1}^{x} \bigl(w^{\odot } \bigr)''(s)\,ds. $$

 □

Note that the sun-dual $Y^{\odot }$ is almost the same as in the book by Diekmann et al. [39, Theorem II.5.2], where it is taken with respect to the first derivative with the condition $\dot{y}(0)=0$. However, in that case there was an extra condition in $Y^{\odot }$ that functions $g \in L^{1}$ could be extended to be zero for $\theta \geq h$. In our case with diffusion we have a fixed domain on which the diffusion takes place, so this condition is not present.

Now we can take the dual again and end up at the dual space $Y^{\odot *}$.

#### Theorem 44

*The dual space* $Y^{\odot *}$ *can be represented as* $\mathbb{R}\times L^{\infty }(\Omega )$. *For the operator* $B^{\odot *}$, *we have that*

103 $$\begin{aligned} D\bigl(B^{\odot *}\bigr) =&\bigl\{ \bigl(\gamma ,w^{\odot *}\bigr)| \bigl(w^{\odot *}\bigr)' \textit{ is Lipschitz continuous}, \\ &{}w^{\odot *}(-1)=\gamma , \bigl(w^{\odot *} \bigr)'( \pm 1)=0 \bigr\} \end{aligned}$$

*and* $B^{\odot *} (\gamma ,w^{\odot *}) := (0, (w^{\odot *})'')$, *where* $(w^{\odot *})''$ *is an* $L^{\infty }(\Omega )$ *function such that*

104 $$ \bigl(w^{\odot *}\bigr)'(x)= \int _{-1}^{x} \bigl(w^{\odot *} \bigr)''(s)\,ds. $$

#### Proof

The dual space of $\mathbb{R}\times L^{1}(\Omega )$ can be represented as $\mathbb{R}\times L^{\infty }(\Omega )$ with the duality pairing between $Y^{\odot *}$ and $Y^{\odot }$ being given by

$$ \bigl\langle \bigl(\gamma , w^{\odot *}\bigr),\bigl(c,w^{\odot } \bigr)\bigr\rangle := \gamma c + \int _{-1}^{1} w^{\odot *}(x)w^{\odot }(x)\,dx. $$

First we prove the ⊆ inclusion of (103). Let $(\gamma ,w^{\odot *})\in D(B^{\odot *})$ and $B^{\odot *}(\gamma ,w^{\odot *})=(\beta ,z^{\odot *})$. Let

$$ v^{\odot *}(x) :=v^{\odot *}(-1)+ \int _{-1}^{x} z^{\odot *}(s)\,ds, $$

which is a Lipschitz continuous function as $z^{\odot *} \in L^{\infty }(\Omega )$. Then, for all $(c,w^{\odot })\in D(B^{\odot })$, we get that

$$\begin{aligned} &\gamma \bigl(w^{\odot }\bigr)'(-1) + \int _{\Omega }w^{\odot *}(x) \bigl(w^{\odot } \bigr)''(x)\,dx \\ &\quad = \bigl\langle \bigl(\gamma ,w^{\odot *}\bigr),B^{\odot }\bigl(c,w^{\odot }\bigr)\bigr\rangle \\ &\quad =\bigl\langle \bigl( \beta ,z^{\odot *}\bigr),\bigl(c,w^{\odot } \bigr)\bigr\rangle \\ &\quad =\beta c + \int _{\Omega }z^{\odot *}(x)w^{\odot }(x)\,dx \\ &\quad =\beta c + v^{\odot *}(x)w^{\odot }(x)|_{-1}^{1} \\ & \qquad {}- \int _{\Omega }v^{\odot *}(x) \bigl(w^{\odot } \bigr)'(x)\,dx \\ &\quad =\beta c + v^{\odot *}(-1)w^{\odot }(x)|_{-1}^{1} + \gamma \bigl(w^{\odot }\bigr)'(-1) \\ &\qquad {}+ \int _{\Omega } \biggl(\gamma + \int _{-1}^{x} v^{\odot *}(s)\,ds \biggr) \bigl(w^{\odot }\bigr)''(x)\,dx. \end{aligned}$$

Here we used that $(w^{\odot })' \in \operatorname{AC}[-1,1]$ and $(w^{\odot })'(1)=0$. As *c* and $w^{\odot }(\pm 1)$ are arbitrary, we see that necessarily $\beta =0$, $v^{\odot *}(\pm 1)=0$. Furthermore,

$$ w^{\odot *}(x)=\gamma + \int _{-1}^{x} v^{\odot *}(s)\,ds, $$

which implies that $(w^{\odot *})'=v^{\odot *}$ and $w^{\odot *}(-1)=\gamma $.

Finally, we prove the ⊇ inclusion of (103). Let $(\gamma ,w^{\odot *})$ be in the right-hand side of (103) and $(c,w^{\odot })\in D(B^{\odot })$. Then, by the calculations above, we get that

$$\begin{aligned} \bigl\langle \bigl(\gamma ,w^{\odot *}\bigr),B^{\odot } \bigl(c,w^{\odot }\bigr)\bigr\rangle &= \gamma \bigl(w^{\odot } \bigr)'(-1) + \int _{\Omega }w^{\odot *}(x) \bigl(w^{\odot } \bigr)''(x)\,dx \\ &= \int _{\Omega }\bigl(w^{\odot *}\bigr)''(x)w^{\odot }(x)\,dx \\ &= \bigl\langle \bigl(0,\bigl(w^{\odot *}\bigr)'' \bigr), \bigl(c,w^{\odot }\bigr)\bigr\rangle , \end{aligned}$$

from which we can conclude that $(\gamma ,w^{\odot *}) \in D(B^{\odot *})$ and $B^{\odot *} (\gamma ,w^{\odot *}) := (0, (w^{\odot *})'')$. □

Finally, we characterise the sun bi-dual $Y^{\odot \odot }$ which is the closure of $D(B^{\odot *})$ with respect to the $Y^{\odot *}$-norm, which is a supremum norm.

#### Theorem 45

*The sun bi*-*dual* $Y^{\odot \odot }$ *can be represented as* $\{(\gamma ,w^{\odot \odot })|w^{\odot \odot }\in C(\Omega ), w^{\odot \odot }(-1)=\gamma \}$. *The canonical embedding* $j_{Y}:Y\rightarrow Y^{\odot *}$ *is given by* $j_{Y} y=(y(-1),y)$. *Moreover*, *Y* *is sun*-*reflexive with respect to the semigroup* *S*, *i*.*e*. $j_{Y}(Y)=Y^{\odot \odot }$.

#### Proof

Let $y^{\odot *} = (\gamma , w^{\odot *}) \in Y^{\odot *}$. As the supremum norm does not preserve derivatives, i.e. the $C^{2}$ functions are dense in $C^{0}$ with respect to the supremum norm, we have that only the continuity and the condition $w^{\odot *}(-1)=\gamma $ remain. For $j_{Y} y=(y(-1),y)$, it can be easily checked that, for any $y^{\odot }\in Y^{\odot }$,

$$ \bigl\langle j_{Y} y, y^{\odot }\bigr\rangle = \bigl\langle y^{\odot }, y\bigr\rangle = \int _{\Omega } \biggl(\gamma + \int _{-1}^{x} v^{\odot *}(s)\,ds \biggr) \bigl(w^{\odot }\bigr)''(x)\,dx =. $$

So $j_{Y}$ is the canonical embedding between *Y* and $Y^{\odot *}$ and it is an isomorphism between *Y* and $Y^{\odot \odot }$. Hence *Y* is sun-reflexive. □

## Appendix B: Proofs

### Lemma 46

*Let* $\Phi , \psi \in L^{\infty }([-h,0];Y^{**})$ *and* $g,\dot{g} \in L^{1}([0,h];Y^{*})$ *such that*

$$\begin{aligned} &\Phi (-t)= \Phi (0) - \int _{0}^{t} \psi (-\theta )\,d\theta, \\ &g(t)= g(0) + \int _{0}^{t} \dot{g}(\theta )\,d\theta \end{aligned}$$

*for all* $t\in [0,h]$, *then it holds that*

$$ \bigl\langle \Phi (-t),g(t) \bigr\rangle = \bigl\langle \Phi (0),g(0) \bigr\rangle + \int _{0}^{t} \bigl\langle \Phi (-\theta ), \dot{g}(\theta ) \bigr\rangle \,d \theta - \int _{0}^{t} \bigl\langle \psi (-\theta ),g( \theta ) \bigr\rangle \,d \theta $$

*for all* $t \in [0,h]$.

### Proof

Let Φ, *ψ*, *g*, *ġ* as above and define the scalar function *ξ*

$$ \xi (t) := \bigl\langle \Phi (-t), g(t) \bigr\rangle $$

for $t \in [0,h]$. As $\Phi \in L^{\infty }([-h,0];Y^{**})$ and $g \in L^{1}([0,h];Y^{*})$, *ξ* is integrable.

By definition *ξ* is absolutely continuous on an interval *I* if, for every $\epsilon >0$, there is $\delta >0$ such that whenever a finite sequence of pairwise disjoint sub-intervals $(s_{k},t_{k})$ of *I* with $t_{k},s_{k}\in I$ satisfies

$$ \sum_{k} (t_{k} - s_{k}) < \delta , $$

then

$$ \sum_{k} \bigl\Vert \xi (t_{k}) - \xi (s_{k}) \bigr\Vert < \epsilon . $$

Both Φ and *g* are absolutely continuous and a.e. differentiable with derivative *ψ* and *ġ* respectively [63, Corollary 1.4.31].

For $t,s\in [0,h]$,

$$\begin{aligned} \bigl\vert \xi (t)-\xi (s) \bigr\vert &= \bigl\vert \bigl\langle \Phi (-t), g(t) \bigr\rangle - \bigl\langle \Phi (-s), g(s) \bigr\rangle \bigr\vert \\ &= \bigl\vert \bigl\langle \Phi (-t) - \Phi (-s), g(t) \bigr\rangle + \bigl\langle \Phi (-s), g(t)-g(s) \bigr\rangle \bigr\vert \\ &\leq \bigl\Vert \Phi (-t) - \Phi (-s) \bigr\Vert \max_{t\in [0,h]} \bigl\Vert g(t) \bigr\Vert + \bigl\Vert g(t) - g(s) \bigr\Vert \max _{t\in [0,h]} \bigl\Vert \Phi (-t) \bigr\Vert . \end{aligned}$$

Hence, by the absolute continuity of Φ and *g*, *ξ* is absolutely continuous and consequently has an a.e. derivative *ξ̇*, which is integrable, and for $t\in [0,h]$

$$ \xi (t) = \xi (0) + \int _{0}^{t} \dot{\xi }(\theta )\,d\theta . $$

Furthermore, we have that

$$ \frac{\xi (t)-\xi (s)}{t-s} = \biggl\langle \Phi (-s), \frac{g(t)-g(s)}{t-s} \biggr\rangle - \biggl\langle \frac{\Phi (-t) - \Phi (-s)}{s-t}, g(t) \biggr\rangle . $$

Taking the limit as $s \rightarrow t$, we can deduce that

$$ \dot{\xi }(t) = \bigl\langle \Phi (-t), \dot{g}(t) \bigr\rangle - \bigl\langle \psi (-t), g(t) \bigr\rangle . $$

Hence we have that, for $t\in [0,h]$,

$$ \bigl\langle \Phi (-t),g(t) \bigr\rangle = \bigl\langle \Phi (0),g(0) \bigr\rangle + \int _{0}^{t} \bigl\langle \Phi (-\theta ), \dot{g}(\theta ) \bigr\rangle \,d \theta - \int _{0}^{t} \bigl\langle \psi (-\theta ),g( \theta ) \bigr\rangle \,d \theta . $$

 □

### Lemma 47

*Define the matrix* $\hat{Q} \in C^{(N+1)\times (N+1)}$ *as*

$$ \hat{Q}_{j,m}= \textstyle\begin{cases} \frac{1}{n_{j}-p_{m}} & \textit{for } j\in \{1,\ldots ,N\}, m\in \{1, \ldots ,N+1\}, \\ 1 & \textit{for } j=N+1, m\in \{1,\ldots ,N+1\}.\end{cases} $$

*When* $n_{i}\neq n_{j}\neq p_{m} \neq p_{l}$ *for* $i,j\in \{1,\ldots ,N\}$, $l,m\in \{1,\ldots ,N+1\}$, $i\neq j$, $l\neq m$, *then* *Q̂* *is invertible*.

### Proof

We subtract the last column from the other columns. We get the following matrix *Q̃*:

$$ \tilde{Q}_{j,m}= \textstyle\begin{cases} \frac{p_{m}-p_{N+1}}{(n_{j}-p_{m})(n_{j}-p_{N+1})} & \text{for } j,m \in \{1,\ldots ,N\}, \\ \frac{1}{n_{j}-p_{N+1}} & \text{for } j\in \{1,\ldots ,N\}, m=N+1, \\ 0 & \text{for } j=N+1, m\in \{1,\ldots ,N\}, \\ 1 & \text{for } j=m=N+1. \end{cases} $$

Now row *j* of matrix *Q̃* contains the factor $\frac{1}{n_{j}-p_{N+1}}$ and column *m* contains the factor $p_{m}-p_{N+1}$ for $j,m \in \{1,\ldots ,N\}$. Hence we can rewrite the determinant of *Q̂* as follows:

$$ \det (\hat{Q})=\det (\tilde{Q})=\det (Q)\prod_{i=1}^{N} \frac{p_{i}-p_{N+1}}{n_{i}-p_{N+1}}. $$

Here matrix $Q \in C^{N\times N}$ is defined as

$$ Q_{j,m}=\frac{1}{n_{j}-p_{m}}\quad \text{for } j,m\in \{1,\ldots ,N \}. $$

We observe that *Q* is a Cauchy matrix when $n_{i}\neq n_{j}\neq p_{m} \neq p_{l}$ for $i,j,l,m\in \{1,\ldots ,N\}$, $i\neq j$, $l\neq m$ and hence invertible. Furthermore, the product $\prod_{i=1}^{N}\frac{p_{i}-p_{N+1}}{n_{i}-p_{N+1}}$ is non-zero, so we conclude that *Q̂* is invertible. □

## Footnotes

1 Note that there is another positive $\lambda \in \mathbb{R}$, not shown in Fig. 3, which solves $\det (S^{\lambda ,\mathrm{odd}})=0$ and $\det (S^{\lambda ,\mathrm{even}})=0$; however, this is a degenerate case as $P^{\lambda }(\rho )$ has a double root. Simulations of the linearised system did not indicate the presence of an unstable mode, so we do not regard this point as an eigenvalue.

## Abbreviations

ODE: Ordinary Differential Equation
ADDE: Abstract Delay Differential Equation
AIE: Abstract Integral Equation
LINP: Linear(ised) Problem
CE: Characteristic Equation
RE: Resolvent Equation
DDE: Delay Differential Equation
